# Regioselective Stepwise Synthesis of Unsymmetrical 1,2,5-Triarylpyrroles via Palladium-Catalyzed Decarboxylative Cross-Coupling and C–H Arylation

**DOI:** 10.3390/molecules31060986

**Published:** 2026-03-15

**Authors:** Cindy Buonomano, Stephanie Patterson, Judith Sorel Ngou, Cynthia Messina, Sarah Taylor, François Bilodeau, Pat Forgione

**Affiliations:** 1 Department of Chemistry and Biochemistry, Concordia University, 7141 Sherbrooke O., Montréal, QC H4B 1R6, Canada; cindy.buonomano@umontreal.ca (C.B.);; 2 Centre in Green Chemistry and Catalysis, Montréal, QC H3C 3J7, Canada; 3 Research and Development, Boehringer Ingelheim (Canada) Ltd., 2100 rue Cunard, Laval, QC H7S 2G5, Canada

**Keywords:** decarboxylative cross-coupling, C–H arylation, pyrroles

## Abstract

Pyrrole derivatives are natural organic molecules that are important to the pharmaceutical industry due to their occurrence in nature and their use in a wide range of medical applications. In general, non-symmetric, 1,2,5-triaryl-substituted pyrroles are prepared either by Paal–Knorr condensation or cycloaddition that present synthetic challenges particularly if late-stage functionalization is required. The present study describes a modular approach to synthesizing 1,2,5-triarylpyrroles containing three different arene substituents. Using pyrrole ester building blocks, a sequence of decarboxylative cross-coupling and C–H arylation provides unsymmetrical 1,2,5-triarylpyrroles in a regioselective, stepwise manner; the scope and limitations of the sequence are disclosed.

## 1. Introduction

**Figure 1:**
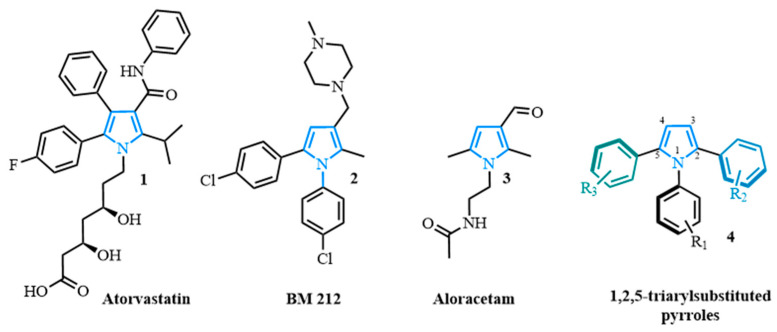
Examples of polysubstituted pyrroles of pharmaceutical interest.

Pyrroles, a class of naturally occurring compounds, and their derivatives have been proven to be therapeutically beneficial [1,2]. Consequently, the pyrrole scaffold is of particular interest to the pharmaceutical industry [3,4] as it can be used to develop anti-cancer agents [5,6], HIV inhibitors [7], and anti-inflammatory compounds [8,9]. The synthesis of pyrrole derivatives has therefore been pursued by several researchers due to their applications (Figure 1) [1,3,10,11,12,13,14,15,16].

Among the wide variety of pyrrole motifs, relatively few 1,2,5-substituted pyrroles have been reported; nevertheless, examples of this scaffold have shown promising biological activity in the context of cardiovascular disease (Atorvastatin **1**) [10,13,14], as potential antimycobacterial agents (BM 212 **2**) [17], or in the treatment of Alzheimer’s disease (Aloracetam **3**) [14] (Figure 1). In particular, 1,2,5-triaryl pyrroles **4** gained initial attention among physical chemists due to their photochemical properties [18,19,20,21,22,23] and, more notably, within the synthetic chemistry community for their pharmaceutical applications [24,25].

The main interest in synthesizing 1,2,5-triaryl-substituted pyrroles lies in the hindered rotation of the N–C(Ar_1_) bond and the two C–C(Ar) bonds (Figure 1), which favors a non-planar arrangement of the three aryl rings, particularly in the presence of ortho substitution. This three-dimensional conformation arises from high torsional barriers about the N–C and C–C bonds, rendering the scaffold conformationally restricted [26,27,28]. As a result, 1,2,5-triaryl-substituted pyrroles can access distinct three-dimensional chemical space relative to coplanar pyrroles, which may translate to improved complementarity with biological targets [29,30]. In some cases, this can be leveraged in atropisomer-based designs without requiring C(sp^3^) stereocentres [31,32,33,34].

**Figure 2:**
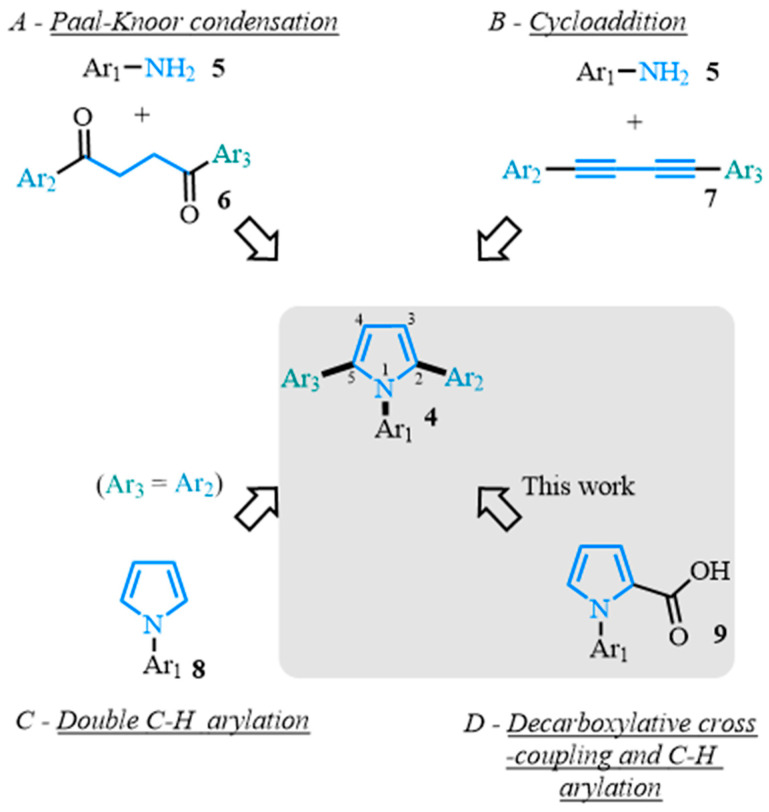
Selected synthetic pathways to 1,2,5-triarylpyrroles. (**A**) Paal-Knoor condensation (**B**) Cycloaddition (**C**) Double C–H arylation (**D**) Decarboxylative cross-coupling and C–H arylation.

Several routes have been reported towards the synthesis of the 1,2,5-triaryl-substituted pyrrole scaffold **4** (Figure 2) [1,3,10,11,12,13,14,16]. Most of the previous methods make use of the pioneering Paal–Knorr [35,36] and Hantzch [37] condensations involving a diketone, **6**, and a primary amine, **5**, respectively (Figure 2A) [38,39,40,41,42,43,44,45,46,47]. Recently, metal-catalyzed cycloadditions of 1,3-butadiynes **7** with primary amines **5** have been developed as a promising alternative approach (Figure 2B) [48,49,50,51,52]. The difficulty of accessing these starting materials limits the routes used to synthesize 1,2,5-triaryl-substituted pyrroles and does not allow for the possibility of late-stage functionalization that is particularly valued in drug discovery and materials applications to fine-tune the desired target properties [38,39,40,41,42,43,44,45,46,47,48,49,50,51,52,53,54,55,56,57,58,59,60]. There have been numerous industrial applications of palladium-catalyzed cross-couplings in the synthesis of therapeutic compounds [61,62,63], providing a more modular strategy. While classic palladium-catalyzed cross-coupling reactions could be employed toward this end, the organometallic derivatives used as coupling partners in the reaction and the release of stoichiometric amounts of organometallic by-products can be challenging to purify and potentially interfere with final applications. Over the past decade, C–H arylation has proven successful as a more atom-economic process in the synthesis of polyarylated pyrroles such as **4** (Figure 2C) [64,65,66,67]. Despite its advantages, the C–H arylation reaction does not allow for regioselective control producing symmetrical molecules [65]. Alternatively, palladium-catalyzed decarboxylative cross-couplings [68,69,70,71,72,73,74,75,76,77] have emerged as a regioselective and sustainable route in the synthesis of heteroaromatics [78,79,80,81,82,83,84,85,86,87] and pyrroles [88], releasing CO_2_ as a by-product. Here we propose a new synthetic pathway towards 1,2,5-triaryl pyrroles **4** featuring palladium-catalyzed decarboxylative cross-coupling and C–H arylation as key steps (Figure 2D) involving a family of N-arylated pyrrole carboxylic acids **9**. Employing decarboxylative cross-coupling followed by C–H arylation ensures a highly regioselective and modular methodology to synthesize 1,2,5-triaryl pyrroles compared to existing procedures.

**Figure 3:**
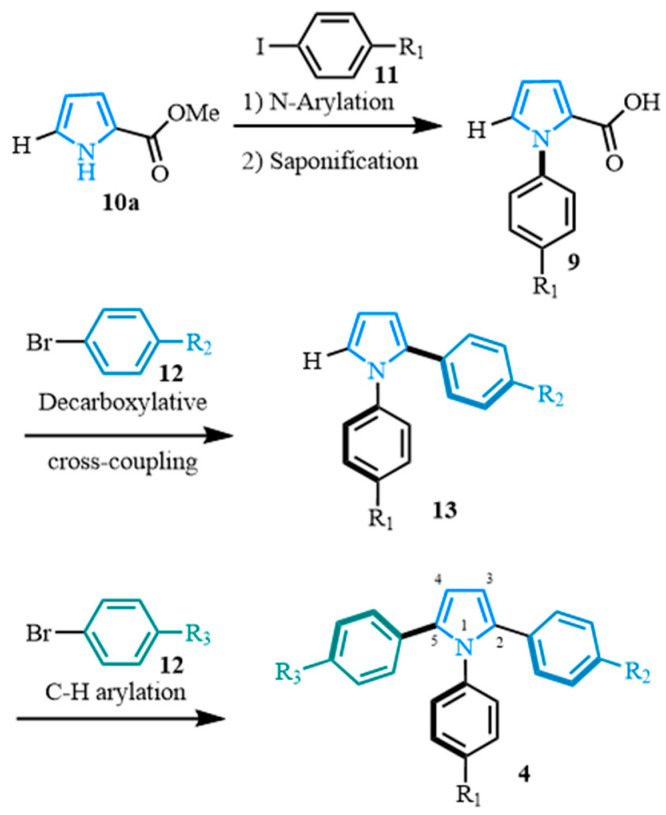
Proposed synthetic pathway toward 1,2,5-triaryl-substituted pyrroles.

Our proposed synthetic pathway starts with the versatile and easily accessible pyrrole ester **10a** (Figure 3). In our approach, various N-arylated pyrrole carboxylic acids **9** were obtained after a Buchwald cross-coupling [89] from the pyrrole carboxylate **10a** and the aryl iodides **11**, followed by a saponification reaction. The carboxylic acids **9** were then submitted to palladium-catalyzed decarboxylative cross-coupling conditions to form the desired diarylated pyrroles **13** [87]. In this way, position two is blocked by the aromatic substituent, controlling the regioselectivity of the subsequent step. The diarylated pyrrole **13** and aryl bromides **12** would then be involved in a C–H arylation [65,67], leading to the non-symmetrical 1,2,5-triaryl pyrroles **4**.

Although the individual transformations employed herein—Pd-catalyzed decarboxylative cross-coupling and Pd-catalyzed C–H arylation of pyrroles—are well established, this work integrates them into an efficient, practical, and regioselective sequence that enables the controlled, sequential installation of three distinct aryl groups at N, C2, and C5 from a common pyrrole-2-carboxylate precursor. Using a modular, building block strategy from commercially available and inexpensive starting materials, the approach facilitates late-stage diversification and provides a regioselective, atom-economic, and tunable route to unsymmetrical 1,2,5-triarylpyrroles that are difficult to access by previous methods.

## 2. Results and Discussion

**Figure 4:**
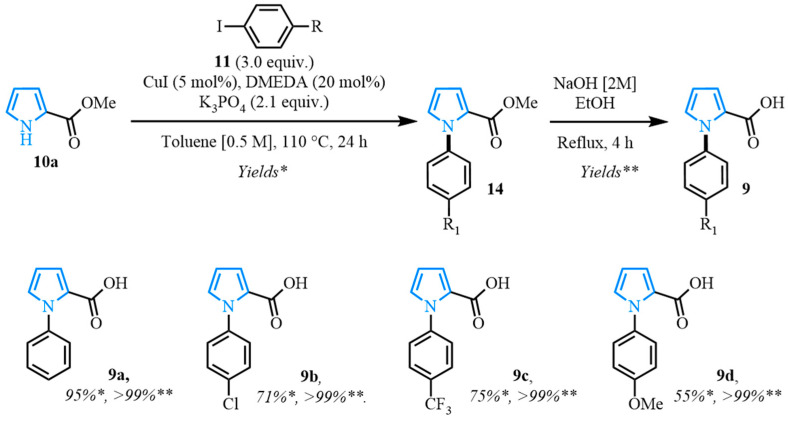
Buchwald coupling and saponification for the synthesis of N-arylated pyrrole carboxylic acids. Yields * corresponds to the yield for Buchwald coupling. Yields ** corresponds to the yield for saponification.

Following a two-step process involving a Buchwald coupling and saponification, four distinct carboxylic acids were obtained (Figure 4). The first arylation involved the preparation of various N-arylated pyrroles that were synthesized from methyl 1*H*-pyrrole-2-carboxylate **10a** using Buchwald’s previously reported conditions [89]. The electron-neutral N-aryl-substituted carboxylic acid **9a** was obtained after a high-yielding C-N coupling reaction was followed by a quantitative saponification. The electron-deficient N-arylated carboxylic acids **9b**–**c** were obtained with good yields, whereas a moderate yield was observed for the formation of the electron-rich N-aryl carboxylic acid **9d**.

After obtaining the carboxylic acids, the palladium-catalyzed decarboxylative cross-coupling step was optimized (Table 1). The optimization was carried out in the presence of the N-phenyl carboxylic acid **9a** and the aryl bromide **12e** to obtain the diarylated pyrrole **13a**. Conditions previously reported [87] were initially evaluated as a starting point for the optimization of the reaction (Table 1, entry 1), producing an excellent yield of the corresponding product.

To improve the efficiency of the cross-coupling, the original 2:1 ratio of acid **9a** towards bromide **12e** was inverted to a 1:2 ratio (Table 1, entry 2), using one equivalent of carboxylic acid **9a** and two equivalents of aryl bromide **12e**. This change resulted in a much lower yield; therefore, the original ratio of two equivalents of carboxylic acid to one equivalent of aryl bromide was used for further optimizations. The reaction was evaluated in the absence of *n*-Bu_4_NCl·H_2_O (Table 1, entry 3), which resulted in the formation of a slurry mixture and gave a yield similar to that obtained with the additive present. However, because the slurry likely led to a less homogeneous reaction medium, potentially increasing by-product formation and lowering yields, further optimizations were performed with the additive included. Continuing the evaluation of the additive impact, reduced amounts of 0.3 and 0.5 equivalents were compared (Table 1, entries 4–5), resulting in similar quantitative yields, with no significant by-product formation or slurry formation. The final optimized conditions used the 0.3 equivalent of *n*-Bu_4_NCl·H_2_O, which was chosen to provide the most atom-economic conditions for the decarboxylative cross-coupling step.

Having determined the optimized conditions, various aryl bromide coupling partners were used to evaluate the scope of the reaction (Table 2). The choice of coupling partners was largely limited to electron-withdrawing groups to maximize the stability of the triaryl pyrroles. Because pyrroles are easily oxidized, electron-rich pyrroles are more susceptible to chemical degradation and can also undergo polymerization under acidic conditions [11,90,91,92,93,94], which can complicate strongly electron-donating substitution patterns in multi-step sequences.

The N-phenyl pyrrole carboxylic acid **9a** was coupled with *para* -NO_2_-, -Me-, and -CF_3_-substituted aryl bromides with excellent isolated yields (Table 2, entries 1–3). The scope of pyrrole carboxylic acids was then extended to additional pyrroles using 4-bromobenzonitrile as the coupling partner (Table 2, entries 4–6). Excellent yields were obtained when electron-deficient carboxylic acids were used (Table 2, entries 4–5). The highest yield was obtained when the pyrrole carboxylic acid was substituted with a -Cl substituent **9b** (Table 2, entry 4) and was marginally lower when pyrrole was substituted with a -CF_3_ group **9c** (Table 2, entry 5). In comparison, electron-rich -OMe-substituted pyrrole **9d** was obtained in a moderate yield (Table 2, entry 6). Overall, a range of diarylated pyrroles **13** were synthesized in moderate to excellent yields.

The final step of the sequence was a C–H arylation based on the conditions derived from Doucet and his research group’s work on related pyrroles (Table 3) [65,67]. We initiated our investigations with pyrrole **13a** and the bromo coupling partner **12h,** leading to the formation of the 1,2,5-triarylated pyrrole **4a** [65,67].

Unfortunately, the previously reported conditions did not result in the expected product **4a** (Table 3, entry 1). Palladium catalyst levels were increased to 5%; however, the expected product still was not observed (Table 3, entry 2). A temperature increase from 150 °C to 170 °C was evaluated that resulted in a moderate yield (Table 3, entry 3). When PPh_3_ was used as the ligand, the yield of the reaction increased significantly (Table 3, entry 4). The stoichiometry between the pyrrole **13a** and the aryl bromide **12h** was assessed, determining if the equivalent of pyrrole could be reduced (Table 3, entry 5). When only one equivalent of pyrrole was used, the results were similar to what was observed when the diarylated species were employed in excess. The pyrrole was maintained as the limiting reagent for further optimization of the C–H arylation reaction. Subsequently, other Pd^II^ and Pd^0^ sources were assessed (Table 3, entries 6–8) where PdCl_2_ was identified as the best palladium source for C–H arylation resulting in an impressive yield (Table 3, entry 6), while the Pd(PPh_3_)_4_ catalyst showed a much lower yield than when generated in situ (Table 3, entry 7). The lower yields were obtained with Pd_2_(dba)_3_ (Table 3, entry 9). After screening different ligands, the bulkier options, such as JohnPhos and CyJohnPhos, were less suitable for this reaction, resulting in lower yields (Table 3, entries 9–10). Finally, while the ligand PCy_3_·HBF_4_ provided the corresponding products in good yields (Table 3, entry 11), the best ligand choice remained PPh_3_. In conclusion, following an evaluation of numerous conditions, the optimal C–H arylation parameters were identified (Table 3, entry 6) for the synthesis of 1,2,5-triaryl pyrroles **4a** in an excellent yield of 92%.

The 1,2-diaryl pyrroles **13** that were synthesized previously were subjected to the optimized conditions for C–H arylation (Table 4) to evaluate the scope of the transformation. First, the effect of the substituent position on the aryl bromide coupling partner was evaluated using diarylated pyrrole **13a** (Table 4, entries 1–3). As previously demonstrated, a *para*-substituted bromo coupling partner can be employed, resulting in excellent yields (Table 4, entry 1). The reaction is also tolerant to substituents present at the *meta*- and *ortho*-positions on the aryl ring, affording the desired product in good yields (Table 4, entries 2–3). A systematic study was carried out employing 1-bromo-4-(trifluoromethyl)benzene **12h** as a coupling partner (Table 4, entries 4–8). The yields were very good in the case of the -NO_2_-substituted pyrrole **13b** (Table 4, entry 4) but were slightly lower for the -CF_3_-substituted pyrrole **13d**, providing moderate yields of the corresponding product (Table 4, entry 5). Lastly, diarylated pyrroles **13** bearing different substituents on the N-phenyl group were evaluated (Table 4, entries 6–8). As previously observed, the electron-deficient-substituted pyrroles **13e**–**f** led to the highest yields (Table 4, entries 6–7) while the electron-rich-substituted pyrrole **13g** provided the lowest yield of their corresponding products (Table 4, entry 6).

**Figure 5:**
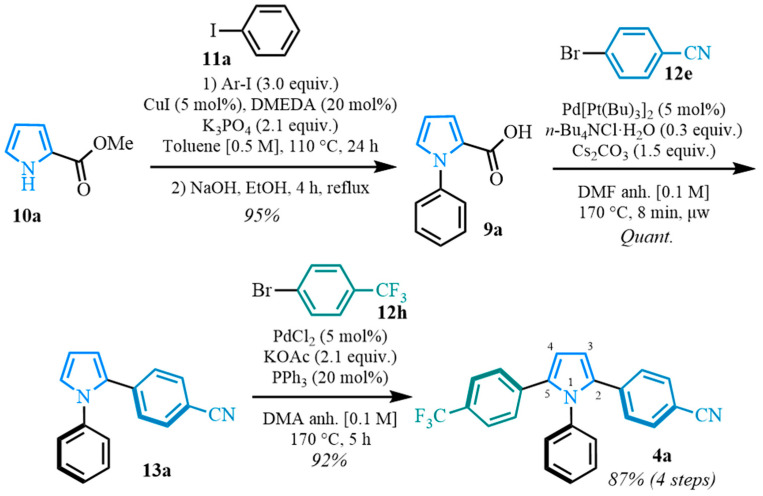
Example of the synthesis of a 1,2,5-triarylated pyrrole with specific yields and conditions.

A representative example is shown in Figure 5 to illustrate the stepwise regiocontrol of the sequence. Although the synthesis comprises four steps overall, it is designed to be modular and provides higher overall yields than previously reported routes. Zhu and their co-workers reported a convergent oxidative route to related triarylpyrroles [55]; in contrast, the present approach enables the programmed, regioselective installation of three distinct aryl groups from readily available aryl halides, which is advantageous for analogue/library synthesis where positional control is required.

## 3. Materials and Methods

### 3.1. General Remarks

Reactions were carried out in regular glassware under air unless otherwise noted. All anhydrous flasks were flame-dried while under high vacuum and purged with argon unless otherwise stated. Commercially available starting materials (methyl 1H-pyrrole-2-carboxylate and all aryl halides) and reagents (K_3_PO_4_, N,N′-Dimethylethylenediamine, CuI, *n*Bu_4_NCl·H_2_O, Cs_2_CO_3_, and K_2_CO_3_) were purchased from Sigma-Aldrich (Burlington, MA, USA), Alfa Aesar (Waltham, MA, USA) and AK Scientific (Union City, CA, USA) and used without further purification. Palladium catalysts were purchased from STREM (Newburyport, MA, USA) and stored under inert gas at room temperature (PdCl_2_) or in the freezer at −10 degrees Celsius (Pd(PtBu_3_)_2_). All solvents were purchased from (Montreal, QC, Canada) and Sigma-Aldrich as ACS grade. Anhydrous solvents were dried and stored in a flame-dried Schlenk flask using 3 Å molecular sieves, which were activated by heating at 150 °C under a high vacuum overnight. Distilled water was obtained from an in-house distillery. Solids were weighed on a balance open to the air and added to a round-bottomed flask or microwave vial unless otherwise noted. Liquids were transferred using a glass syringe with a stainless-steel needle or a micropipette for µL volumes unless noted otherwise. Compounds were purified using column chromatography on silica-gel (Zeoprep 60 Eco, 40–63 µm, and Zeochem AG (Rüti, Switzerland)) from SiliCycle (Quebec City, QC, Canada). Microwave-assisted reactions were carried out using the Biotage Initiator™ 2.3 build 6250 microwave system with a 400 W magnetron (Biotage, Uppsala, Sweden), which monitors temperature during the reaction time period and adjusts wattage accordingly. Proton nuclear magnetic resonance spectra (^1^H NMR) were measured at 500 MHz using a Varian VNMRS-500 (Varian, Inc., Palo Alto, CA, USA) in CDCl_3_ unless stated otherwise. Carbon–nuclear magnetic resonance spectra (^13^C NMR) were measured at 125 MHz using the Varian VNMRS-500 in CDCl_3_ unless stated otherwise. The chemical shifts are reported in parts per million (ppm) and referenced from either residual solvent or the tetramethylsilane (TMS) signal. The multiplicity is represented as: s = singlet, d = doublet, t = triplet, q = quartet and m = multiplet, which is indicated in parentheses along with the number of protons and coupling constants (in Hz). High-resolution mass spectral data (HRMS) was collected using a LC-TOF ESI mass spectrometer (Agilent, Santa Clara, CA, USA) operated in positive ion mode unless otherwise noted. Yield determination was completed using ^1^H NMR and was done using trimethoxybenzene (TMB) as an internal standard. ^1^H NMR and ^13^C NMR spectra are available in the Supplementary Materials.

### 3.2. Synthetic Procedures

#### 3.2.1. General Procedure for the Buchwald Couplings of Methyl 1H-Pyrrole-2-carboxylate (A)

To a 5 mL conical microwave vial that was equipped with a spin-vein added methyl 1H-pyrrole-2-carboxylate (2.0 mmol, 1.0 equiv.), the aryliodide (6.0 mmol, 3.0 equiv.), K_3_PO_4_ (4.2 mmol, 2.1 equiv.), N,N′-Dimethylethylenediamine (0.4 mmol, 20 mol%), and CuI (0.1 mmol, 5 mol%). Next, 2 mL of anhydrous Toluene were added to the vial. The reaction vial was immersed into a preheated oil bath at 110 °C and heated for 24 h. After cooling down, the crude mixture was diluted with ethyl acetate (10 mL) and water (10 mL). The organic layer was washed with a saturated NaCl aqueous solution (2 × 10 mL). The combined aqueous phases were washed with EtOAc (3 × 10 mL). The combined organic phases were dried over Na_2_SO_4_, and after filtration the solvent evaporated under reduced pressure, and the solid residue was purified by flash column chromatography.

#### 3.2.2. General Procedure for the Formation of Carboxylic Acids (B)

The pyrrole ester (2 mmol, 1.0 equiv.) was dissolved in ethanol (1 mL). A solution of NaOH 2M (20 mmol, 10 equiv.) was added to the mixture. The reaction was heated at reflux temperature for 4 h and then allowed to cool down. The ethanol was evaporated under vacuo. The crude reaction media was diluted with ethyl acetate (10 mL) and water (10 mL). The organic layer was discarded. Ethyl acetate was added to the separatory funnel (10 mL). The media was acidified to pH = 2 using a pure solution of hydrochloric acid. The aqueous layer was washed with ethyl acetate (2 × 10 mL). The combined organic layers were washed with a saturated NaCl aqueous solution (10 mL). The combined organic phases were dried over Na_2_SO_4_ and after filtration the solvent evaporated under reduced pressure and the solid residue was collected.

#### 3.2.3. General Procedure for the Palladium-Catalyzed Decarboxylative Cross-Couplings (C)

To a 10 mL conical microwave vial equipped with a spin-vein was added the pyrrole carboxylic acid (2.0 mmol, 2.0 equiv.), the aryl coupling partner (1.0 mmol, 1.0 equiv.), *n*Bu_4_NCl·H_2_O (0.6 mmol, 0.3 equiv.), Cs_2_CO_3_ (3.0 mol, 1.5 equiv.), and Pd[P(tBu)_3_]_2_ (0.10 mmol, 5 mol%). 5 mL of anhydrous DMF were added to the vial. The reaction vial was pre-stirred for 30 s at 23 °C followed by 8 min of heating at 170 °C. After cooling down, the crude mixture was diluted with ethyl acetate (50 mL) and water (50 mL). The organic layer was washed with a saturated NaHCO_3_ aqueous solution (2 × 20 mL), a saturated NaCl aqueous solution (2 × 20 mL). The combined aqueous phases were washed with EtOAc (3 × 20 mL). The combined organic phases were dried over Na_2_SO_4_ and after filtration the solvent evaporated under reduced pressure and the solid residue was purified by flash column chromatography.

#### 3.2.4. General Procedure for the Palladium-Catalyzed C–H Arylations (D)

A 5 mL conical microwave vial equipped with a spin-vane was added with the diaryl pyrrole (0.2 mmol, 1.0 equiv.), the aryl coupling partner (0.4 mmol, 2.0 equiv.), KOAc (0.4 mmol, 2.0 equiv.), PPh_3_ (0.04 mol, 20 mol%), and PdCl_2_ (0.01 mmol, 5 mol%). Then, 2 mL of anhydrous DMA were added to the vial. The reaction vial was immersed into a preheated oil bath at 170 °C and heated for 5 h. After cooling down, the crude mixture was diluted with ethyl acetate (10 mL) and water (10 mL). The organic layer was washed with a saturated NaHCO_3_ aqueous solution (2 × 10 mL) and a saturated NaCl aqueous solution (2 × 10 mL). The combined aqueous phases were washed with EtOAc (3 × 10 mL). The combined organic phases were dried over Na_2_SO_4_, and after filtration the solvent evaporated under reduced pressure and the solid residue were purified by flash column chromatography.

### 3.3. Spectroscopic Data

methyl 1-phenyl-1*H*-pyrrole-2-carboxylate (**14a**). Following the general procedure (A), methyl 1*H*-pyrrole-2-carboxylate (2.5 g, 20 mmol) was reacted with iodobenzene (60 mmol). The spectral data was in agreement with reported data [95].

methyl 1-(4-chlorophenyl)-1*H*-pyrrole-2-carboxylate (**14b**). Following the general procedure (A), methyl 1*H*-pyrrole-2-carboxylate (1.25 g, 10 mmol) was reacted with 1-chloro-4-iodobenzene (30 mmol). The spectral data was in agreement with reported data [95].

methyl 1-(4-(trifluoromethyl)phenyl)-1*H*-pyrrole-2-carboxylate (**14c**). Following the general procedure (A), methyl 1*H*-pyrrole-2-carboxylate (2.5 g, 20 mmol) was reacted with 1-iodo-4-(trifluoromethyl)benzene (60 mmol). The spectral data was in agreement with reported data [95].

methyl 1-(4-methoxyphenyl)-1*H*-pyrrole-2-carboxylate (**14d**). Following the general procedure (A), methyl 1*H*-pyrrole-2-carboxylate (2.5 g, 20 mmol) was treated with 1-iodo-4-methoxybenzene (60 mmol). The spectral data was in agreement with reported data [95].

1-phenyl-1*H*-pyrrole-2-carboxylic acid (**9a**). Following the general procedure (B), methyl 1-phenyl-1*H*-pyrrole-2-carboxylate (3.8 g, 19 mmol) was reacted with sodium hydroxide (190 mmol). After extraction, 1-phenyl-1*H*-pyrrole-2-carboxylic acid (9a) was obtained as a white powder (3.55 g, 100%). The spectral data was in agreement with reported data [95].

1-(4-chlorophenyl)-1*H*-pyrrole-2-carboxylic acid (**9b**). Following the general procedure (B), methyl 1-(4-chlorophenyl)-1*H*-pyrrole-2-carboxylate (1.7 g, 7.1 mmol) was reacted with sodium hydroxide (71 mmol). After extraction, 1-(4-chlorophenyl)-1*H*-pyrrole-2-carboxylic acid (**9b**) was obtained as a brown powder (1.55 g, 99%). The spectral data was in agreement with reported data [95].

1-(4-(trifluoromethyl)phenyl)-1*H*-pyrrole-2-carboxylic acid (**9c**). Following the general procedure (B), methyl 1-(4-(trifluoromethyl)phenyl)-1*H*-pyrrole-2-carboxylate (4.0 g, 15 mmol) was reacted with sodium hydroxide (150 mmol). After extraction, 1-(4-(trifluoromethyl)phenyl)-1*H*-pyrrole-2-carboxylic acid (**9c**) was obtained as a white powder (3.8 g, 98%). The spectral data was in agreement with reported data [95].

1-(4-methoxyphenyl)-1*H*-pyrrole-2-carboxylic acid (**9d**). Following the general procedure (B), methyl 1-(4-methoxyphenyl)-1*H*-pyrrole-2-carboxylate (2.5 g, 11 mmol) was reacted with sodium hydroxide (110 mmol). After extraction, 1-(4-methoxyphenyl)-1*H*-pyrrole-2-carboxylic acid (9d) was obtained as a pink powder (2.32 g, 97%). The spectral data was in agreement with reported data [95].

4-(1-phenyl-1*H*-pyrrol-2-yl)benzonitrile (**13a**). Following the general procedure (C), 1-phenyl-1*H*-pyrrole-2-carboxylic acid (0.8 mmol, 149 mg) was reacted with 1-bromo-4-(trifluoromethyl)benzene (0.4 mmol). The reaction was purified by flash chromatography (9:1 hexanes/diethyl ether) to afford 4-(1-phenyl-1H-pyrrol-2-yl)benzonitrile as a white powder (0.36 mmol, 89%). The spectral data was in agreement with reported data [96].

2-(4-nitrophenyl)-1-phenyl-1*H*-pyrrole (**13b**). Following the general procedure (C), 1-phenyl-1*H*-pyrrole-2-carboxylic acid (0.8 mmol, 149 mg) was reacted with 1-bromo-4-nitrobenzene (0.4 mmol). The reaction was purified by flash chromatography (9:1 hexanes/diethyl ether) to afford 2-(4-nitrophenyl)-1-phenyl-1*H*-pyrrole (0.36 mmol, 90%) as yellow crystals. Rf: 0.44 (90:10 hexanes/diethyl ether). ^1^H NMR (300 MHz, CDCl_3_) δ = 8.05 (d, *J* = 8.9 Hz and 2H), 7.39 (m, 3H), 7.25–7.15 (m, 4H), 7.02 (dd, *J* = 2.8, 1.7 Hz and 1H), 6.63 (dd, *J* = 3.7, 1.7 Hz and 1H), 6.41 (dd, *J* = 3.7, 2.8 Hz and 1H) ppm. ^13^C NMR (75 MHz, CDCl_3_) δ = 145.58, 139.96, 139.24, 131.48, 129.56, 129.55, 127.89, 127.71, 127.03, 126.89, 125.80, 125.79, 123.59, 113.36, 110.06 ppm. The spectral data was in agreement with reported data [96].

1-phenyl-2-(p-tolyl)-1*H*-pyrrole (**13c**). Following the general procedure (C), 1-phenyl-1*H*-pyrrole-2-carboxylic acid (0.8 mmol, 149 mg) was reacted with 1-bromo-4-methylbenzene (0.4 mmol). The reaction was purified by flash chromatography (9:1 hexanes/diethyl ether) to afford 1-phenyl-2-(p-tolyl)-1*H*-pyrrole (0.37 mmol, 93%) as a white powder. Rf: 0.32 (90:10 hexanes/diethyl ether). ^1^H NMR (300 MHz, CDCl_3_) δ = 7.65 (d, *J* = 2.0 Hz and 1H), 7.61–7.46 (m, 5H), 7.45–7.37 (m, 3H), 7.17 (dd, *J* = 2.8, 1.8 Hz and 1H), 6.64 (d, *J* = 1.8 Hz and 1H), 6.61–6.55 (m, 1H), 2.54 (s, 3H) ppm. ^13^C NMR (75 MHz, CDCl_3_) δ = 140.63, 135.96, 133.89, 130.12, 128.83, 128.23, 126.53, 125.74, 124.09, 124.00, 110.32, 110.19, 109.19, 99.99, 21.12 ppm. HRMS (ESI): calculated for [C17H15N + H] = 234.1277, found 234.1277. The spectral data was in agreement with reported data [97].

1-phenyl-2-(4-(trifluoromethyl)phenyl)-1*H*-pyrrole (**13d**). Following the general procedure (C), 1-phenyl-1*H*-pyrrole-2-carboxylic acid (0.8 mmol, 149 mg) was reacted with 1-bromo-4-(trifluoromethyl)benzene (0.4 mmol). The reaction was purified by flash chromatography (9:1 hexanes/diethyl ether) to afford 1-phenyl-2-(4-(trifluoromethyl)phenyl)-1*H*-pyrrole (0.36 mmol, 89%) as a white powder. Rf: 0.24 (90:10 hexanes/diethyl ether). ^1^H NMR (300 MHz, CDCl_3_) δ = 7.44 (d, *J* = 8.2 Hz and 2H), 7.38–7.28 (m, 3H), 7.21 (d, *J* = 8.2 Hz and 2H), 7.17 (dd, *J* = 8.1, 1.6 Hz and 2H), 6.98 (dd, *J* = 2.8, 1.8 Hz and 1H), 6.53 (dd, *J* = 3.6, 1.8 Hz and 1H), 6.39 (dd, *J* = 3.6, 2.8 Hz and 1H) ppm. ^13^C NMR (75 MHz, CDCl_3_) δ = 140.18, 136.38, 132.26, 129.26, 128.16, 128.01, 127.73, 127.04, 126.07, 125.70, 125.57, 125.09, 125.05, 122.47, 111.98, 109.69, 109.59 ppm. HRMS (ESI): calculated for [C_17_H_12_F_3_N + H] = 288.0995, found 288.0997. The spectral data was in agreement with reported data [98].

4-(1-(4-chlorophenyl)-1*H*-pyrrol-2-yl)benzonitrile (**13e**). Following the general procedure (C), 1-(4-chlorophenyl)-1*H*-pyrrole-2-carboxylic acid (2.0 mmol, 443 mg) was reacted with 1-bromo-4-(trifluoromethyl)benzene (1.0 mmol). The reaction was purified by flash chromatography (95:5 hexanes/diethyl ether) to afford 4-(1-(4-chlorophenyl)-1*H*-pyrrol-2-yl)benzonitrile (0.93 mmol, 93%) as a white powder. Rf: 0.42 (95:5 hexanes/diethyl ether). ^1^H NMR (300 MHz, CDCl_3_) δ = 7.54–7.42 (m, 2H), 7.39–7.29 (m, 2H), 7.23–7.16 (m, 2H), 7.14–7.04 (m, 2H), 6.96 (dd, *J* = 2.8, 1.7 Hz and 1H), 6.55 (dd, *J* = 3.7, 1.8 Hz and 1H), 6.40 (dd, *J* = 3.7, 2.8 Hz and 1H) ppm. ^13^C NMR (75 MHz, CDCl_3_) δ = 138.52, 136.97, 133.09, 132.17, 131.82, 129.66, 128.01, 126.88, 126.29, 118.94, 113.14, 110.38, 109.55 ppm. HRMS (ESI): calculated for [C_17_H_11_ClN_2_] = 278.0605, found 278.0605.

4-(1-(4-(trifluoromethyl)phenyl)-1*H*-pyrrol-2-yl)benzonitrile (**13f**). Following the general procedure (C), 1-(4-(trifluoromethyl)phenyl)-1*H*-pyrrole-2-carboxylic acid (0.2 mmol, 51 mg) was reacted with 1-bromo-4-(trifluoromethyl)benzene (0.1 mmol). The reaction was purified by flash chromatography (9:1 hexanes/diethyl ether) to afford 4-(1-(4-(trifluoromethyl)phenyl)-1*H*-pyrrol-2-yl)benzonitrile (0.09 mmol, 83%) as a white powder. Rf: 0.13 (9:1 hexanes/diethyl ether). ^1^H NMR (300 MHz, CDCl_3_) δ = 7.68–7.58 (m, 2H), 7.57–7.47 (m, 2H), 7.31–7.23 (m, 2H), 7.19 (d, *J* = 8.5 Hz and 2H), 7.01 (dd, *J* = 2.9, 1.7 Hz and 1H), 6.58 (dd, *J* = 3.7, 1.7 Hz and 1H), 6.44 (dd, *J* = 3.7, 2.9 Hz and 1H) ppm. ^13^C NMR (75 MHz, CDCl_3_) δ = 142.83, 136.81, 132.26, 132.09, 131.87, 129.43, 128.24, 128.13, 126.64, 126.23, 126.12, 125.68, 125.53, 121.91, 118.84, 113.81, 110.80, 109.82 ppm. HRMS (ESI): calculated for [C_18_H_11_F_3_N_2_] = 312.0869, found 312.0867.

4-(1-(4-methoxyphenyl)-1*H*-pyrrol-2-yl)benzonitrile (**13g**). Following the general procedure (C), 1-(4-methoxyphenyl)-1*H*-pyrrole-2-carboxylic acid (2.0 mmol, 434 mg) was reacted with 1-bromo-4-(trifluoromethyl)benzene (1.0 mmol). The reaction was purified by flash chromatography (9:1 hexanes/diethyl ether) to afford 4-(1-(4-methoxyphenyl)-1*H*-pyrrol-2-yl)benzonitrile (0.55 mmol, 55%) as a white powder. Rf: 0.18 (9:1 hexanes/diethyl ether). ^1^H NMR (300 MHz, CDCl_3_) δ = 7.46 (d, *J* = 8.5 Hz and 2H), 7.24–7.14 (m, 2H), 7.12–7.05 (m, 2H), 6.94 (dd, *J* = 2.8, 1.7 Hz and 1H), 6.93–6.84 (m, 2H), 6.55 (dd, *J* = 3.7, 1.7 Hz and 1H), 6.36 (dd, *J* = 3.7, 2.8 Hz and 1H), 3.83 (s, 3H) ppm. ^13^C NMR (75 MHz, CDCl_3_) δ = 158.71, 137.36, 133.07, 131.87, 127.93, 127.00, 126.60, 119.14, 114.49, 112.19, 109.53, 109.06, 55.61 ppm. HRMS (ESI): calculated for [C_18_H_14_N_2_O + H] = 275.1179, found 275.1178.

4-(1-phenyl-5-(4-(trifluoromethyl)phenyl)-1*H*-pyrrol-2-yl)benzonitrile (**4a**). Following the general procedure (D), 4-(1-phenyl-1*H*-pyrrol-2-yl)benzonitrile (0.2 mmol, 49 mg) was reacted with 1-bromo-4-(trifluoromethyl)benzene (0.4 mmol). The reaction was purified by flash chromatography (9:1 hexanes/diethyl ether) to afford 4-(1-phenyl-5-(4-(trifluoromethyl)phenyl)-1H-pyrrol-2-yl)benzonitrile (0.18 mmol, 90%) as light orange crystals. Rf: 0.27 (9:1 hexanes/diethyl ether). ^1^H NMR (500 MHz, CDCl3) δ = 7.43 (t, J = 8.1 Hz and 4H), 7.40–7.27 (m, 3H), 7.13 (d, J = 8.2 Hz and 2H), 7.11 (d, J = 8.3 Hz and 2H), 7.04 (dd, J = 7.2, 1.9 Hz and 2H), 6.61 (d, J = 3.8 Hz and 1H), 6.58 (d, J = 3.8 Hz and 1H) ppm. 13C NMR (126 MHz, CDCl3) δ = 138.05, 137.24, 134.56, 131.80, 129.44, 128.63, 128.54, 128.47, 128.29, 124.96, 118.94, 112.08, 111.63, 109.56 ppm. HRMS (ESI): calculated for [C_24_H_15_F_3_N_2_] = 388.1182, found 388.1179. The spectral data was in agreement with reported data [55].

4-(1-phenyl-5-(3-(trifluoromethyl)phenyl)-1*H*-pyrrol-2-yl)benzonitrile (**4b**). Following the general procedure (D), 4-(1-phenyl-1*H*-pyrrol-2-yl)benzonitrile (0.1 mmol, 25 mg) was reacted with 1-bromo-3-(trifluoromethyl)benzene (0.2 mmol). The reaction was purified by flash chromatography (9:1 hexanes/diethyl ether) to afford 4-(1-phenyl-5-(3-(trifluoromethyl)phenyl)-1*H*-pyrrol-2-yl)benzonitrile (0.09 mmol, 90%) as white crystals. Rf: 0.25 (9:1 hexanes/diethyl ether). ^1^H NMR (500 MHz, CDCl_3_) δ = 7.44 (d, *J* = 8.5 Hz and 2H), 7.41 (d, *J* = 7.8 Hz and 1H), 7.37–7.27 (m, 5H), 7.20 (d, *J* = 7.9 Hz and 1H), 7.13 (d, *J* = 8.5 Hz and 2H), 7.04 (dd, *J* = 8.3, 1.5 Hz and 2H), 6.62 (d, *J* = 3.8 Hz and 1H), 6.57 (d, *J* = 3.8 Hz and 1H) ppm. ^13^C NMR (126 MHz, CDCl_3_) δ = 138.00, 137.25, 135.91, 133.31, 131.81, 131.64, 129.40, 128.64, 128.43, 128.41, 128.27, 125.30, 118.96, 111.99, 111.22, 109.49 ppm. HRMS (ESI): calculated for [C_24_H_15_F_3_N_2_] = 388.1182, found 388.1180.

4-(1-phenyl-5-(2-(trifluoromethyl)phenyl)-1*H*-pyrrol-2-yl)benzonitrile (**4c**). Following the general procedure (D), 4-(1-phenyl-1*H*-pyrrol-2-yl)benzonitrile (0.1 mmol, 25 mg) was reacted with 1-bromo-2-(trifluoromethyl)benzene (0.2 mmol). The reaction was purified by flash chromatography (9:1 hexanes/diethyl ether) to afford 4-(1-phenyl-5-(2-(trifluoromethyl)phenyl)-1*H*-pyrrol-2-yl)benzonitrile (0.08 mmol, 85%) as white crystals. Rf: 0.21 (9:1 hexanes/diethyl ether). ^1^H NMR (500 MHz, CDCl_3_) δ =7.70–7.64 (m, 1H), 7.43 (d, *J* = 8.5 Hz and 2H), 7.33 (t, *J* = 7.7 Hz and 1H), 7.28 (dd, *J* = 7.7, 1.3 Hz and 1H), 7.19 (dd, *J* = 6.3, 1.5 Hz and 3H), 7.14 (d, *J* = 8.5 Hz and 2H), 7.00 (d, *J* = 7.6 Hz and 1H), 6.98–6.93 (m, 2H), 6.63 (d, *J* = 3.7 Hz and 1H), 6.46 (dd, *J* = 3.8, 1.0 Hz and 1H) ppm. ^13^C NMR (126 MHz, CDCl_3_) δ = 138.23, 137.44, 133.81, 133.11, 131.82, 130.51, 128.95, 128.40, 128.00, 127.84, 127.65, 125.99, 119.08, 112.42, 111.46, 109.11 ppm. HRMS (ESI): calculated for [C_24_H_15_F_3_N_2_ + H] = 389.1260, found 389.1258.

2-(4-nitrophenyl)-1-phenyl-5-(4-(trifluoromethyl)phenyl)-1*H*-pyrrole (**4d**). Following the general procedure (D), 2-(4-nitrophenyl)-1-phenyl-1*H*-pyrrole (0.4 mmol, 106 mg) was reacted with 1-bromo-4-(trifluoromethyl)benzene (0.8 mmol). The reaction was purified by flash chromatography (9:1 hexanes/diethyl ether) to afford 2-(4-nitrophenyl)-1-phenyl-5-(4-(trifluoromethyl)phenyl)-1*H*-pyrrole (0.33 mmol, 83%) as yellow crystals. Rf: 0.42 (9:1 hexanes/diethyl ether). ^1^H NMR (500 MHz, CDCl_3_) δ = 8.07–7.98 (m, 2H), 7.44 (d, *J* = 8.2 Hz and 2H), 7.41–7.29 (m, 3H), 7.19–7.14 (m, 4H), 7.10–7.03 (m, 2H), 6.68 (d, *J* = 3.8 Hz and 1H), 6.59 (d, *J* = 3.8 Hz and 1H) ppm. ^13^C NMR (126 MHz, CDCl_3_) δ = 145.74, 139.18, 138.02, 136.32, 135.99, 134.20, 129.53, 128.63, 128.58, 128.42, 128.31, 125.02, 124.99, 124.96, 123.46, 112.68, 111.79 ppm. HRMS (ESI): calculated for [C_23_H_15_F_3_N_2_O_2_ + H] = 409.1158, found 409.1156.

1-phenyl-2,5-bis(4-(trifluoromethyl)phenyl)-1*H*-pyrrole (**4e**). Following the general procedure (D), 1-phenyl-2-(4-(trifluoromethyl)phenyl)-1*H*-pyrrole (0.25 mmol, 71 mg) was reacted with 1-bromo-4-(trifluoromethyl)benzene (0.5 mmol). The reaction was purified by flash chromatography (100 hexanes) to afford 1-phenyl-2,5-bis(4-(trifluoromethyl)phenyl)-1*H*-pyrrole (0.13 mmol, 53%) as white crystals. Rf: 0.21 (100 hexanes). ^1^H NMR (500 MHz, CDCl_3_) δ = 7.42 (d, *J* = 8.2 Hz and 4H), 7.38–7.27 (m, 3H), 7.14 (d, *J* = 8.2 Hz and 4H), 7.08–7.02 (m, 2H), 6.57 (s, 2H) ppm. ^13^C NMR (126 MHz, CDCl_3_) δ = 138.21, 136.29, 135.15, 129.31, 128.70, 128.46, 128.07, 124.95, 124.92, 111.40 ppm. HRMS (ESI): calculated for [C_24_H_15_F_6_N] = 431.1103, found 431.1108. The spectral data was in agreement with reported data [64].

4-(1-(4-chlorophenyl)-5-(4-(trifluoromethyl)phenyl)-1*H*-pyrrol-2-yl)benzonitrile (**4f**). Following the general procedure (D), 4-(1-(4-chlorophenyl)-1*H*-pyrrol-2-yl)benzonitrile (0.4 mmol, 111 mg) was reacted with 1-bromo-4-(trifluoromethyl)benzene (0.8 mmol). The reaction was purified by flash chromatography (9:1 hexanes/diethyl ether) to afford 4-(1-(4-chlorophenyl)-5-(4-(trifluoromethyl)phenyl)-1*H*-pyrrol-2-yl)benzonitrile (0.28 mmol, 71%) as white crystals. Rf: 0.16 (9:1 hexanes/diethyl ether). ^1^H NMR (500 MHz, CDCl_3_) δ = 7.48 (t, *J* = 8.4 Hz and 4H), 7.30 (d, *J* = 8.6 Hz and 2H), 7.13 (dd, *J* = 10.0, 8.4 Hz and 4H), 6.97 (d, *J* = 8.6 Hz and 2H), 6.60 (d, *J* = 3.8 Hz and 1H), 6.57 (d, *J* = 3.8 Hz and 1H) ppm. ^13^C NMR (126 MHz, CDCl_3_) δ = 136.94, 136.54, 135.81, 134.52, 134.16, 131.97, 129.76, 129.69, 128.65, 128.58, 125.14, 118.80, 112.41, 111.96, 109.90 ppm. HRMS (ESI): calculated for [C_24_H_14_ClF_3_N_2_ + H] = 423.0870, found 423.0870.

4-(1,5-bis(4-(trifluoromethyl)phenyl)-1*H*-pyrrol-2-yl)benzonitrile (**4g**). Following the general procedure (D), 4-(1-(4-(trifluoromethyl)phenyl)-1*H*-pyrrol-2-yl)benzonitrile (0.4 mmol, 125 mg) was reacted with 1-bromo-4-(trifluoromethyl)benzene (0.8 mmol). The reaction was purified by flash chromatography (9:1 hexanes/diethyl ether) to afford 4-(1,5-bis(4-(trifluoromethyl)phenyl)-1*H*-pyrrol-2-yl)benzonitrile (0.26 mmol, 66%) as white crystals. Rf: 0.18 (9:1 hexanes/diethyl ether). ^1^H NMR (500 MHz, CDCl_3_) δ = 7.59 (d, *J* = 8.3 Hz and 2H), 7.48 (t, *J* = 8.3 Hz and 4H), 7.13 (dd, *J* = 12.2, 8.2 Hz and 4H), 7.10 (d, *J* = 8.4 Hz and 2H), 6.62 (d, *J* = 3.8 Hz and 1H), 6.59 (d, *J* = 3.8 Hz and 1H) ppm. ^13^C NMR (126 MHz, CDCl_3_) δ = 141.07, 136.79, 135.79, 135.64, 134.54, 132.02, 130.37, 130.11, 129.07, 128.91, 128.81, 128.72, 128.68, 126.53, 125.24, 124.59, 122.92, 122.43, 118.69, 112.82, 112.38, 110.14 ppm. HRMS (ESI): calculated for [C_25_H_14_F_6_N_2_ + H] = 419.1366, found 419.1364.

4-(1-(4-methoxyphenyl)-5-(4-(trifluoromethyl)phenyl)-1*H*-pyrrol-2-yl)benzonitrile (**4h**). Following the general procedure (D), 4-(1-(4-methoxyphenyl)-1*H*-pyrrol-2-yl)benzonitrile (0.4 mmol, 109 mg) was reacted with 1-bromo-4-(trifluoromethyl)benzene (0.8 mmol). The reaction was purified by flash chromatography (9:1 hexanes/diethyl ether) to afford 4-(1-(4-methoxyphenyl)-5-(4-(trifluoromethyl)phenyl)-1*H*-pyrrol-2-yl)benzonitrile (0.2 mmol, 45%) as white crystals. Rf: 0.12 (9:1 hexanes/diethyl ether). ^1^H NMR (500 MHz, CDCl_3_) δ = 7.45 (dd, *J* = 8.6, 7.2 Hz and 4H), 7.15 (dd, *J* = 10.9, 8.3 Hz and 4H), 6.96 (d, *J* = 8.8 Hz and 2H), 6.83 (d, *J* = 8.8 Hz and 2H), 6.60 (d, *J* = 3.8 Hz and 1H), 6.56 (d, *J* = 3.8 Hz and 1H), 3.83 (s, 3H) ppm. ^13^C NMR (126 MHz, CDCl_3_) δ = 159.18, 137.33, 136.17, 135.98, 134.67, 131.82, 130.81, 129.59, 128.49, 128.47, 128.41, 124.97, 118.99, 114.54, 111.81, 111.37, 109.44, 55.44 ppm. HRMS (ESI): calculated for [C_25_H_17_F_3_N_2_O] = 456.1056, found 456.1054.

## 4. Conclusions

We have described a flexible and modular method that uses several key reactions, including Pd-catalyzed decarboxylative cross-coupling and C–H arylation, to efficiently synthesize 1,2,5-triarylpyrroles. Key advantages include simple catalytic systems, short reaction times, practical protocols, and readily available starting materials. Owing to the modularity of the sequence, a range of 1,2,5-triarylpyrroles was prepared. Scope studies focused on aryl bromides to prioritize analogue synthesis and consistency across the sequence since aryl bromides are well documented coupling partners for both the decarboxylative cross-coupling and C–H arylation steps. Because the two steps were optimized under different solvent/base/ligand conditions, the process was carried out stepwise; telescoping with intermediate workup and solvent exchange is a potential direction for future development. These compounds exhibit potentially interesting properties that arise from their three-dimensional shape, which allows them to occupy new chemical space and may enhance interactions with biological targets. Given established atropisomerism in substituted aryl–heteroarene frameworks, the unsymmetrical substitution patterns accessible here may also serve as a starting point for future studies of conformationally restricted or atropisomeric scaffolds.

## Figures and Tables

**Table 1:** Optimization of the decarboxylative cross-coupling.

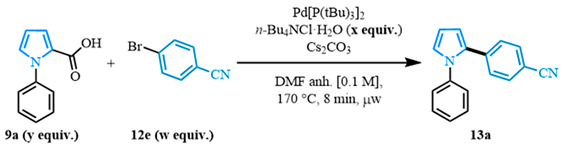			
Entry	9a:12e Ratio (y Equiv.:w Equiv.)	*n*-Bu_4_NCl·H_2_O (x Equiv.)	^1^H NMR Yield (%)
1	2:1	1.0	93 (89)
2	1:2 ^a^	1.0	31
3	2:1	0	85–95
4	2:1	0.3	Quant. (95)
5	2:1	0.5	Quant.

General conditions: 2.0 equiv. of 1-phenyl-*1H*-pyrrole-2-carboxylic acid (0.2 mmol), 1.0 equiv. of 4-bromobenzonitrile, 1.5 equiv. of Cs_2_CO_3_, *n*-Bu_4_NCl·H_2_O, Pd[P(*t*Bu)_3_]_2_ (5 mol%) and 2 mL of anhydrous DMF heated for 8 min at 170 °C in the microwave. **Conditions a:** 1.0 equiv. of 1-phenyl-*1H*-pyrrole-2-carboxylic acid (0.1 mmol), 2.0 equiv. of 4-bromobenzonitrile, 1.5 equiv. of Cs_2_CO_3_, *n*-Bu_4_NCl·H_2_O, Pd[P(*t*Bu)_3_]_2_ (5 mol%) and 2 mL of anhydrous DMF heated for 8 min at 170 °C in the microwave. Yield determined by ^1^H NMR with 1,3,5-trimethoxybenzene as the internal standard. The yields in parenthesis are isolated.

**Table 2:** Scope of the decarboxylative cross-coupling.

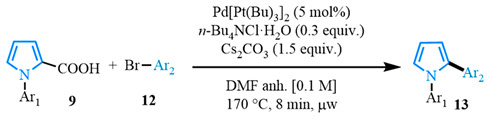			
Entry	Carboxylic Acid	Product	Yield (%)
1	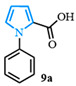	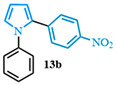	90
2	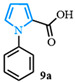	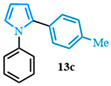	93
3	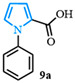	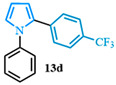	89
4	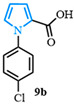	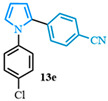	93
5	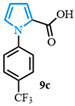	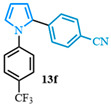	83
6	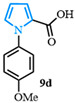	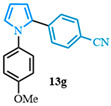	55

General conditions: 2.0 equiv. of 1-phenyl-*1H*-pyrrole-2-carboxylic acid (0.2 mmol), 1.0 equiv. of 4-bromobenzonitrile, 1.5 equiv. of Cs_2_CO_3_, 0.3 equiv. of *n*-Bu_4_NCl·H_2_O, Pd[P(*t*Bu)_3_]_2_ (5 mol%) and 2 mL of anhydrous DMF heated for 8 min at 170 °C in the microwave.

**Table 3:** Optimization of the C–H arylation.

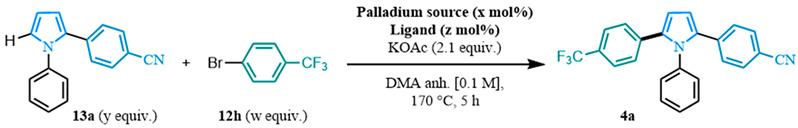							
Entry	Palladium Source		Ligand		Temperature (°C)	A:B Ratio	^1^H NMR Yield (%)
	Source	(x mol%)	Source	(z mol%)			
1	Pd(OAc)_2_	0.1	-	-	150	2:1	0
2	Pd(OAc)_2_	5	-	-	150	2:1	0
3	Pd(OAc)_2_	5	-	-	170	2:1	47
4	Pd(OAc)_2_	5	PPh_3_	20	170	2:1	74
5	Pd(OAc)_2_	5	PPh_3_	20	170	1:2 ^b^	74
6	PdCl_2_	5	PPh_3_	20	170	1:2 ^b^	92
7	Pd(PPh_3_)_4_	5	-	-	170	1:2 ^b^	58
8	Pd_2_(dba)_3_	5	-	-	170	1:2 ^b^	38
9	PdCl_2_	5	JohnPhos	10	170	1:2 ^b^	75
10	PdCl_2_	5	CyJohnPhos	10	170	1:2 ^b^	68
11	PdCl_2_	5	PCy_3_·HBF_4_	10	170	1:2 ^b^	81

General conditions: 2.0 equiv. of methyl 1-phenyl-*1H*-pyrrole-2-carboxylate (0.2 mmol), 1.0 equiv. of 1-bromo-4-(trifluoromethyl)benzene, 2.1 equiv. of KOAc, palladium source (x mol%), ligand (20 mol%) and 2 mL of anhydrous DMA heated for 5 h. **Conditions b**: 1.0 equiv. of methyl 1-phenyl-*1H*-pyrrole-2-carboxylate (0.1 mmol), 2.0 equiv. of 1-bromo-4-(trifluoromethyl)benzene, 2.1 equiv. of KOAc, Pd(OAc)_2_ (5 mol%), PPh_3_ (20 mol%) and 2 mL of anhydrous DMA heated for 5 h. The yield was determined by ^1^H NMR with 1,3,5-trimethoxybenzene as internal standard.

**Table 4:** Scope of the C–H arylation.

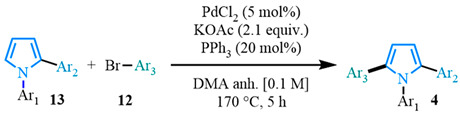			
Entry	Carboxylic Acid	Product	Yield (%)
1	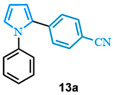	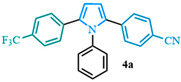	92
2	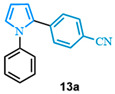	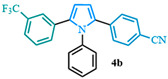	90
3	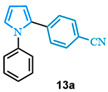	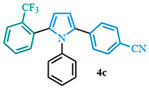	85
4	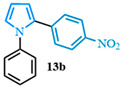	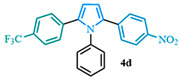	83
5	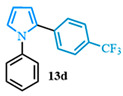	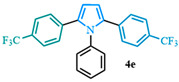	53
6	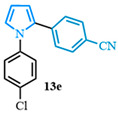	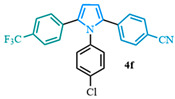	71
7	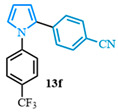	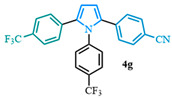	66
8	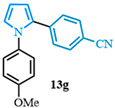	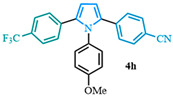	45

General conditions: 1.0 equiv. of pyrrole (0.4 mmol), 2.0 equiv. of aryl bromide, 2.1 equiv. of KOAc, PdCl_2_ (5 mol%), PPh_3_ (20 mol%) and 2 mL of anhydrous DMA heated for 5 h.

## Data Availability

Dataset available on request from the authors.

## Supplementary Materials

The following supporting information can be downloaded at https://www.mdpi.com/article/10.3390/molecules31060986/s1. File S1: ^1^HNMR and ^13^CNMR spectra are available.

## Abbreviations

The following abbreviations are used in this manuscript:

DMEDA	N,N′-Dimethylethylenediamine
DMF	Dimethylformamide
DMA	Dimethylacetamide
NMR	Nuclear magnetic resonance
HRMS	High-resolution mass spectrometry
TMS	Tetramethylsilane
TMB	1,3,5-Trimethoxybenzene
