# Supplementary material for: Regioselective Stepwise Synthesis of Unsymmetrical 1,2,5-Triarylpyrroles via Palladium-Catalyzed Decarboxylative Cross-Coupling and C–H Arylation
Source: Molecules. 2026 Mar 15;31(6):986. doi: 10.3390/molecules31060986 (PMC13029164; doi:10.3390/molecules31060986)

Supplementary Materials

Regioselective Stepwise Synthesis of Unsymmetrical 1,2,5-Triarylpyrroles via Palladium-Catalyzed Decarboxylative Cross-Coupling and C–H Arylation

Cindy Buonomano [1,2], Stephanie Patterson [1], Judith Sorel Ngou [1], Cynthia Messina [1], Sarah Taylor [1], François Bilodeau [3] and Pat Forgione [1,2,*]

[1]Department of Chemistry and Biochemistry, Concordia University, 7141 Sherbrooke O., Montréal, QC H4B 1R6, Canada; cindy.buonomano@umontreal.ca (C.B.)

[2]Centre in Green Chemistry and Catalysis, Montréal, QC H3C 3J7, Canada

[3]Research and Development, Boehringer Ingelheim (Canada) Ltd., 2100 rue Cunard, Laval, QC H7S 2G5, Canada

*Correspondence: pat.forgione@concordia.ca

Table of Contents

1. ^{1}HNMR and ^{13}CNMR spectra 2

1. ^{1}HNMR and ^{13}CNMR spectra

^{1}HNMR of **14a** (300 MHz in $CDCl_3$)

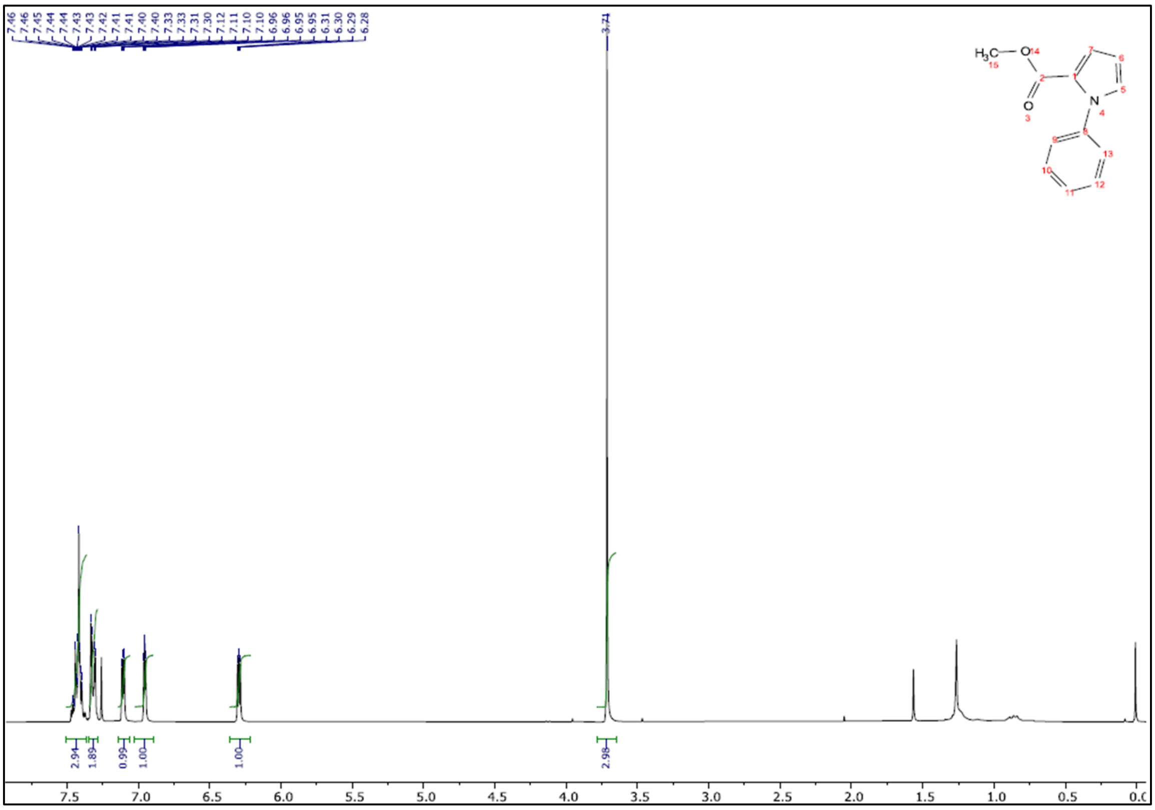

^{1}HNMR of **14b** (300 MHz in $CDCl_3$)

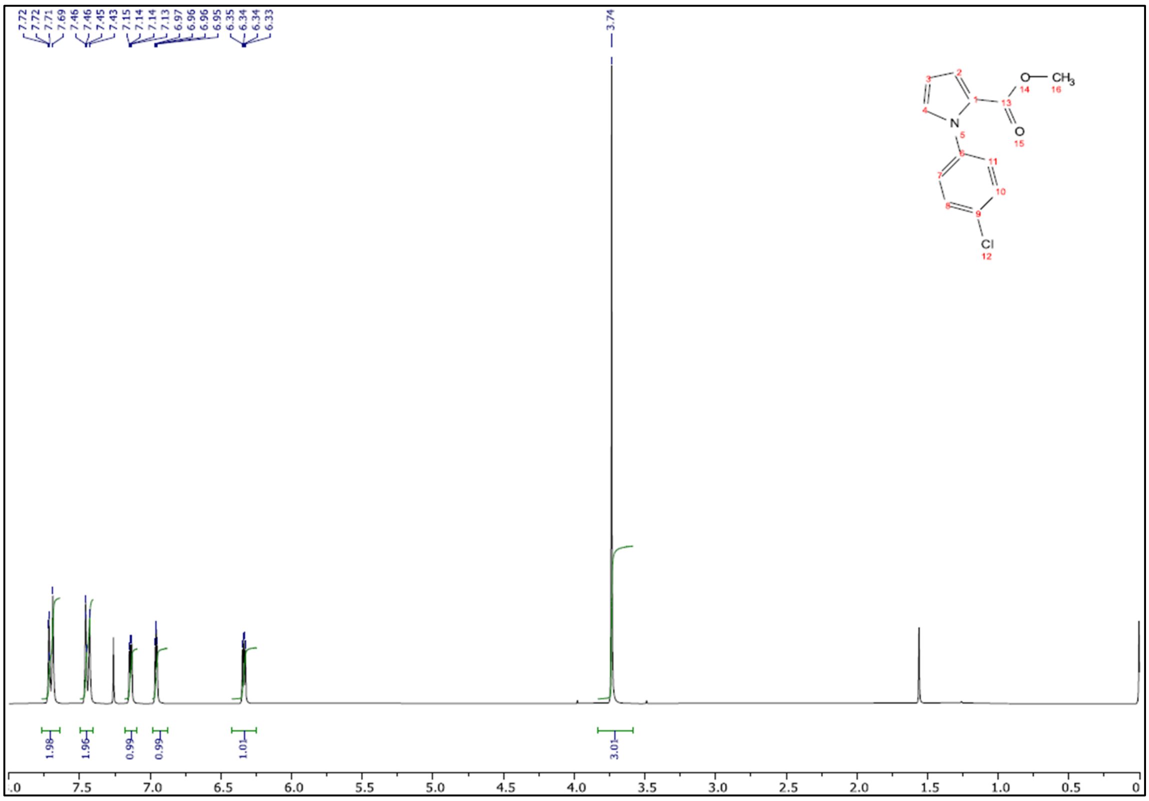

^{1}HNMR of **14c** (300 MHz in $CDCl_3$)

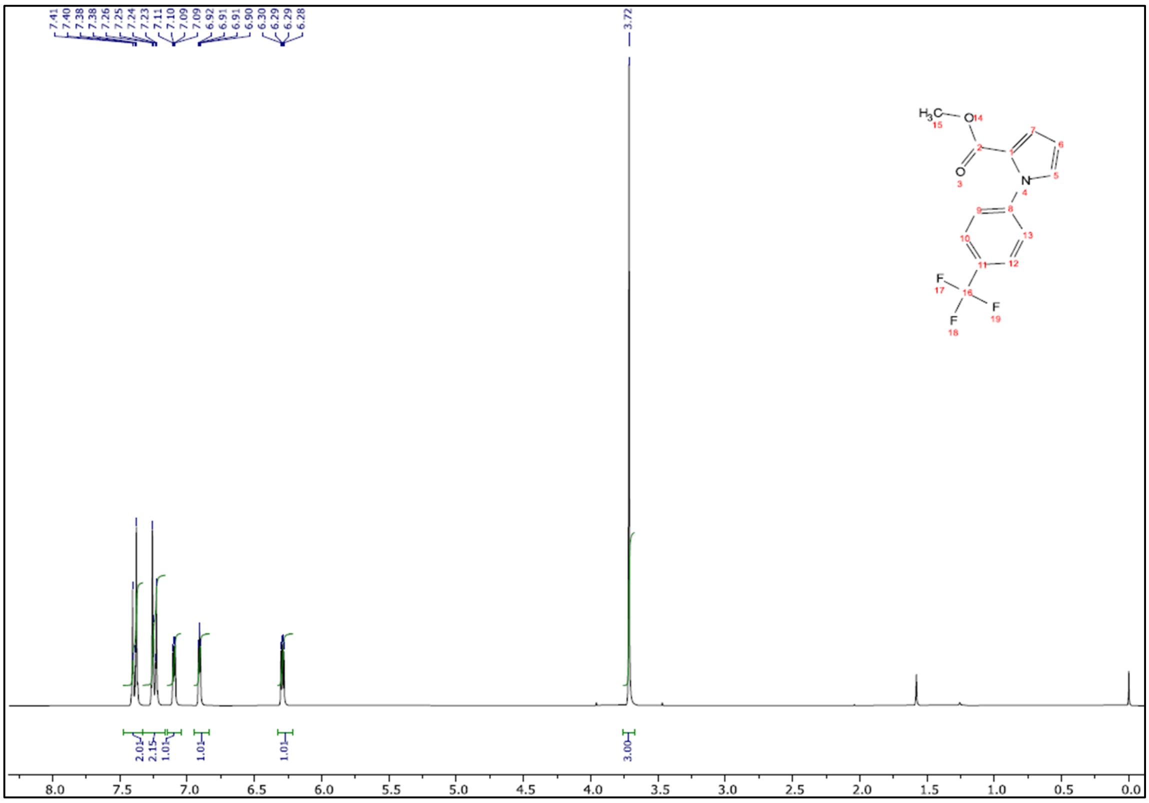

^{1}HNMR of **14d** (300 MHz in $CDCl_3$)

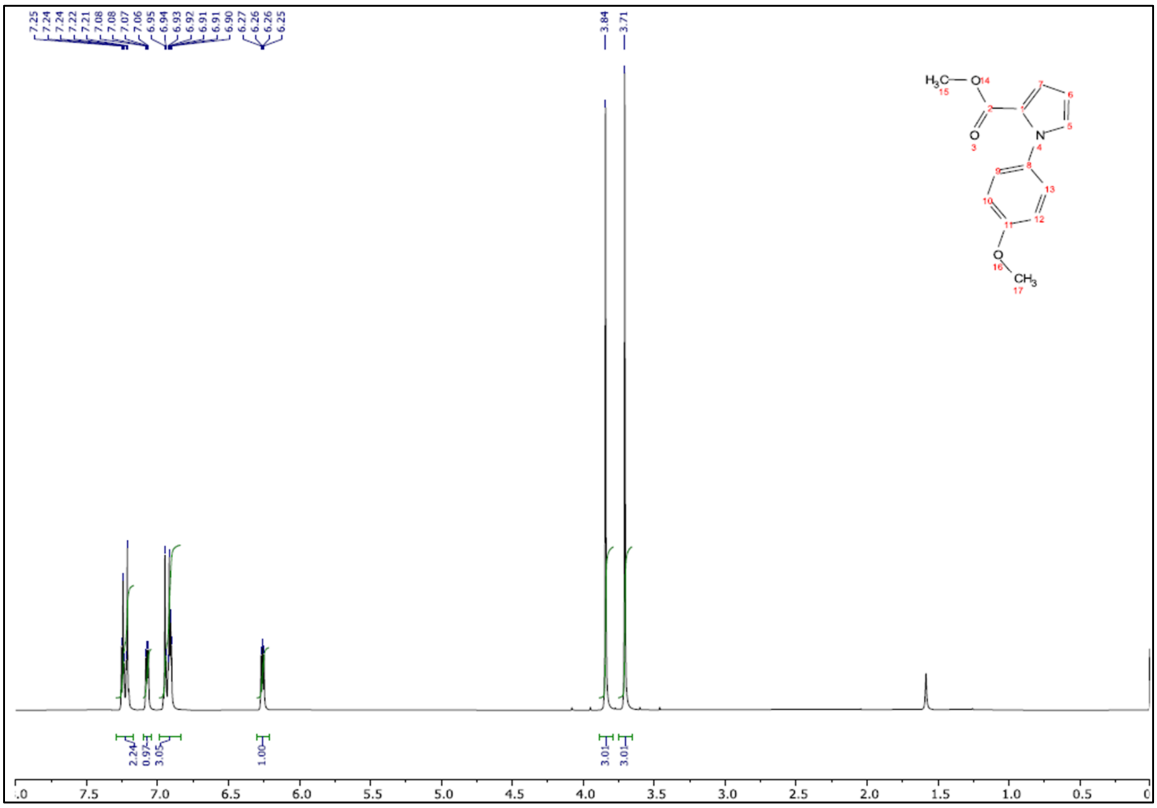

^{1}HNMR of **9a** (300 MHz in DMSO-*d6*)

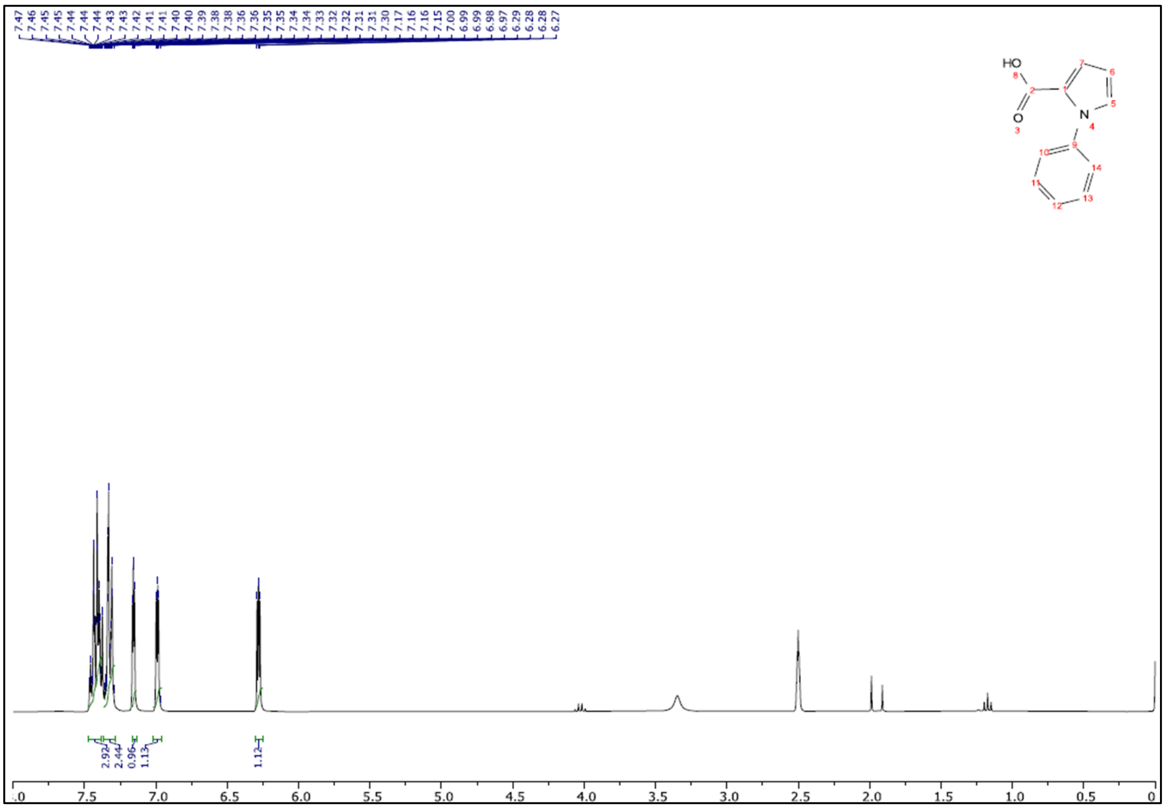

^{1}HNMR of **9b** (300 MHz in DMSO-*d6*)

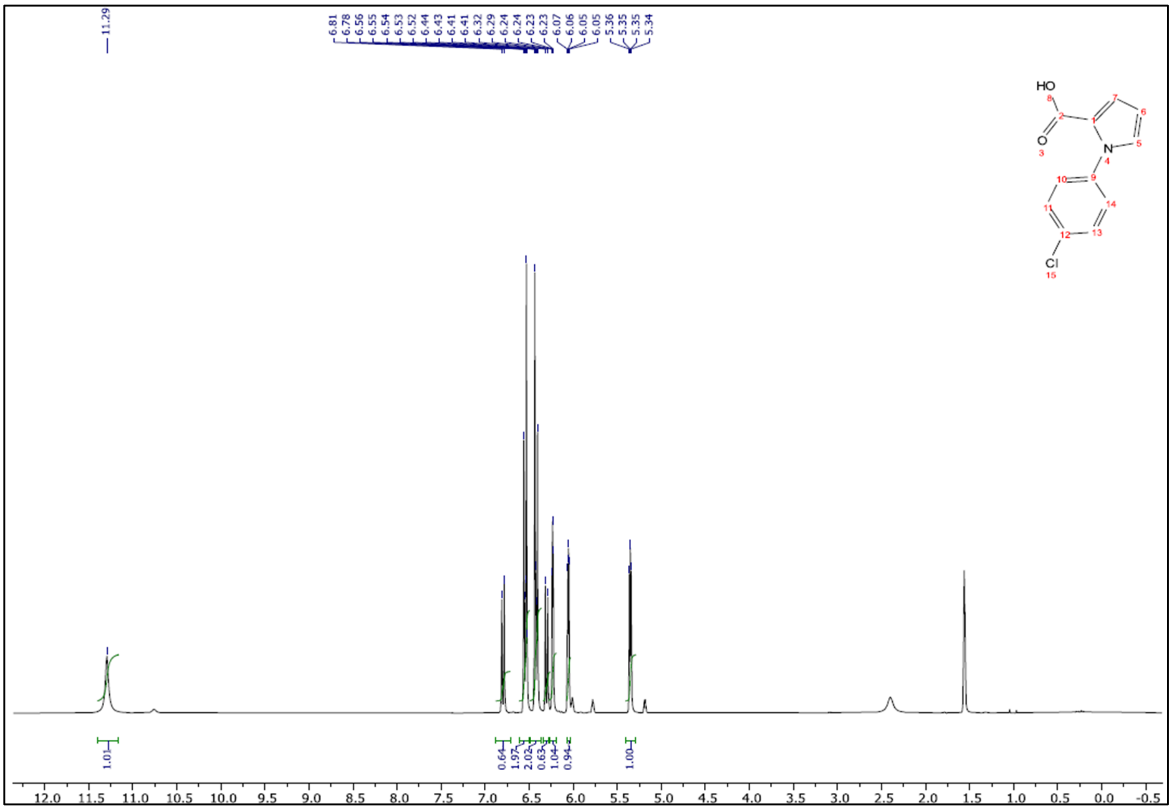

^{1}HNMR of **9c** (300 MHz in DMSO-*d6*)

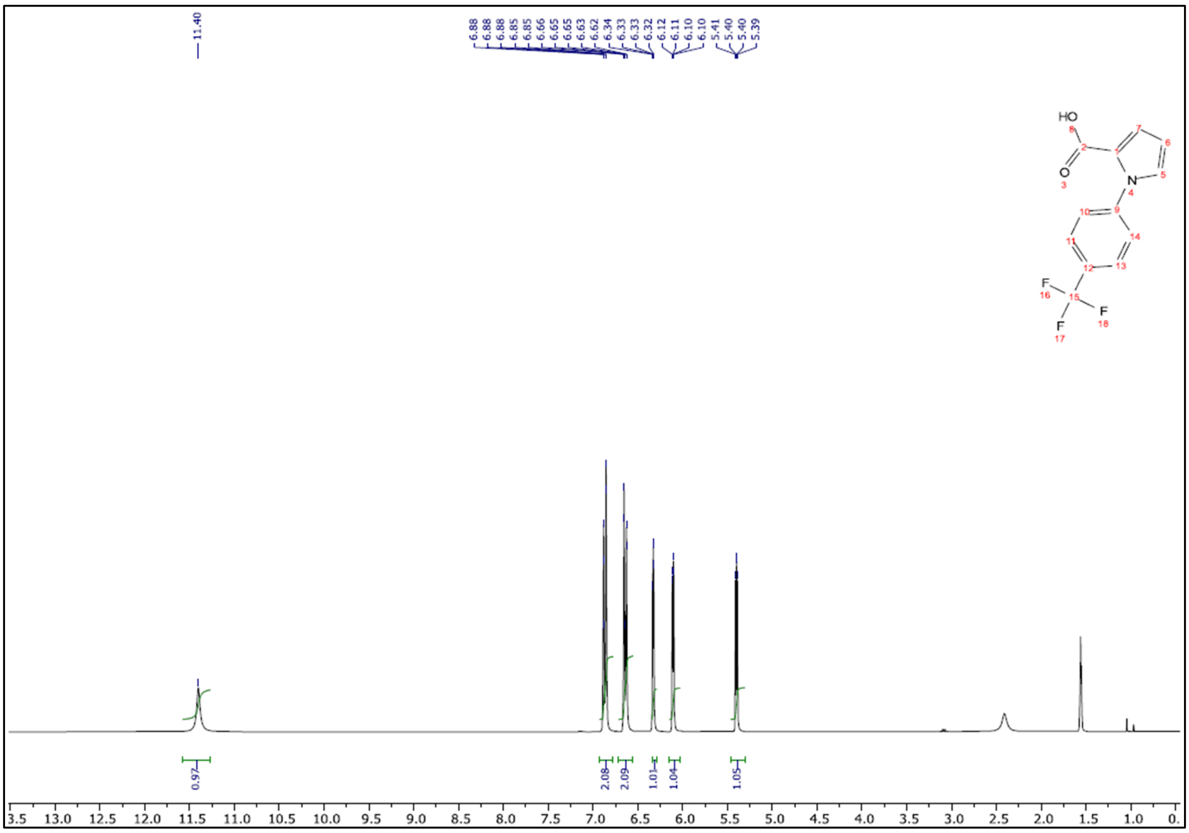

^{1}HNMR of **9d** (300 MHz in DMSO-*d6*)

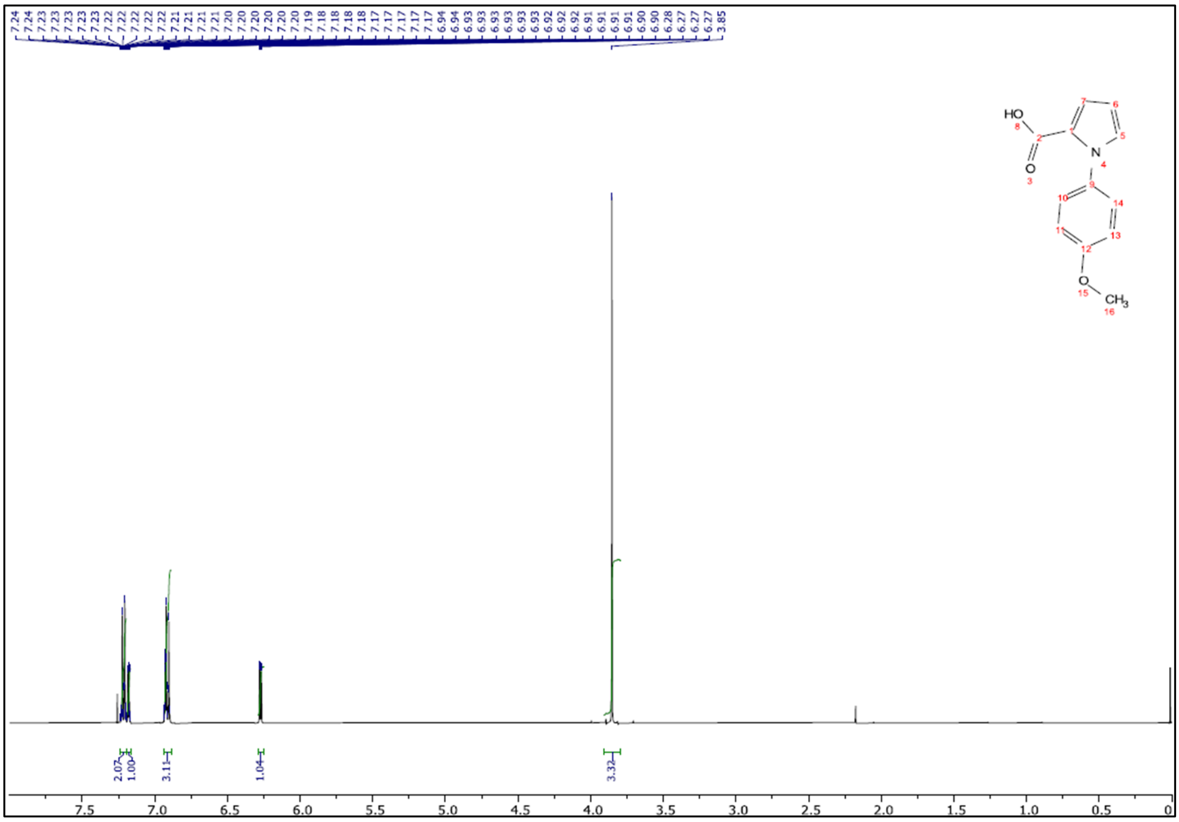

^{1}HNMR of **13a** (300 MHz in $CDCl_3$)

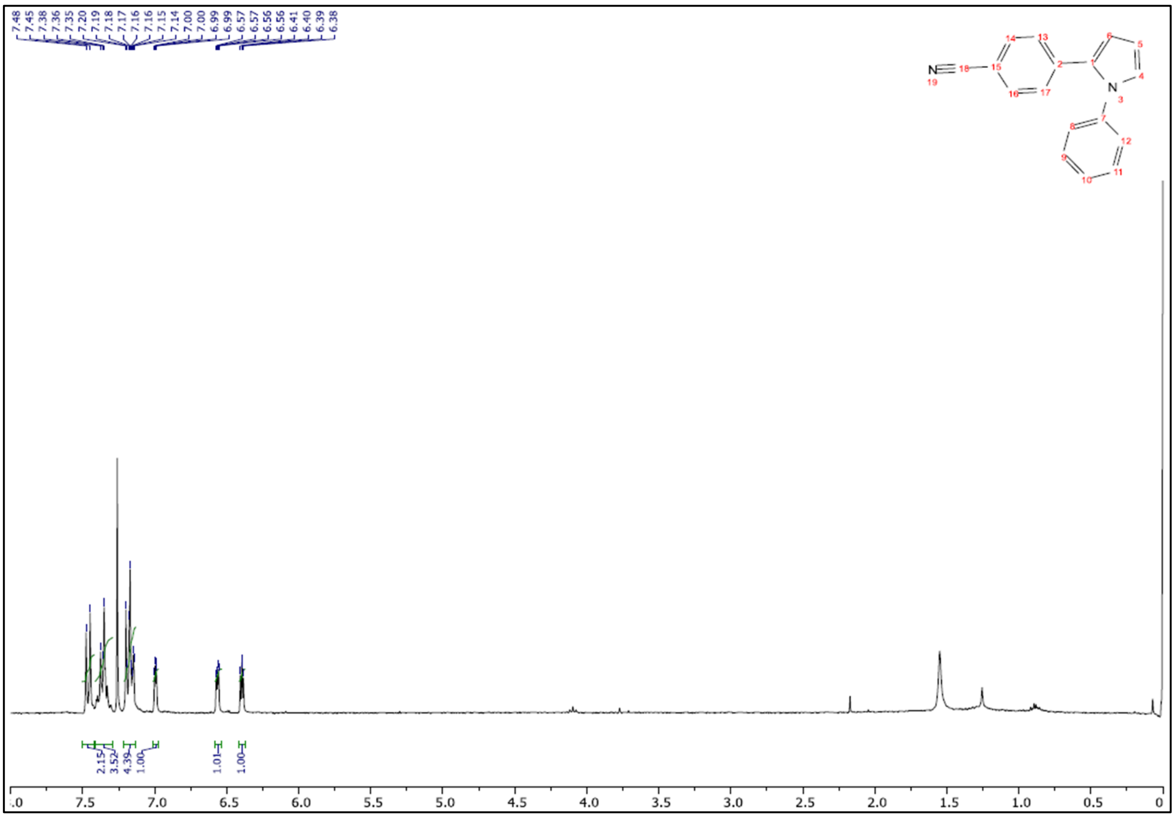

^{1}HNMR of **13b** (300 MHz in $CDCl_3$)

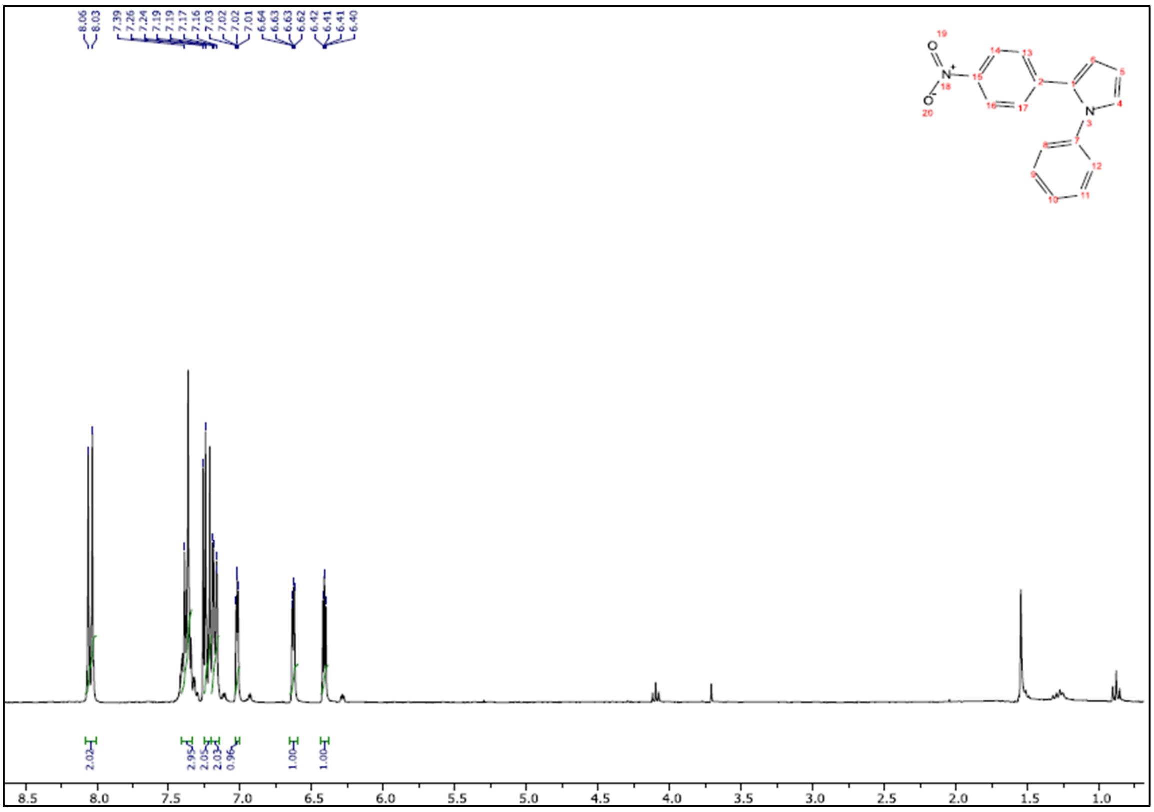

^{13}CNMR of **13b** (75 MHz in $CDCl_3$)

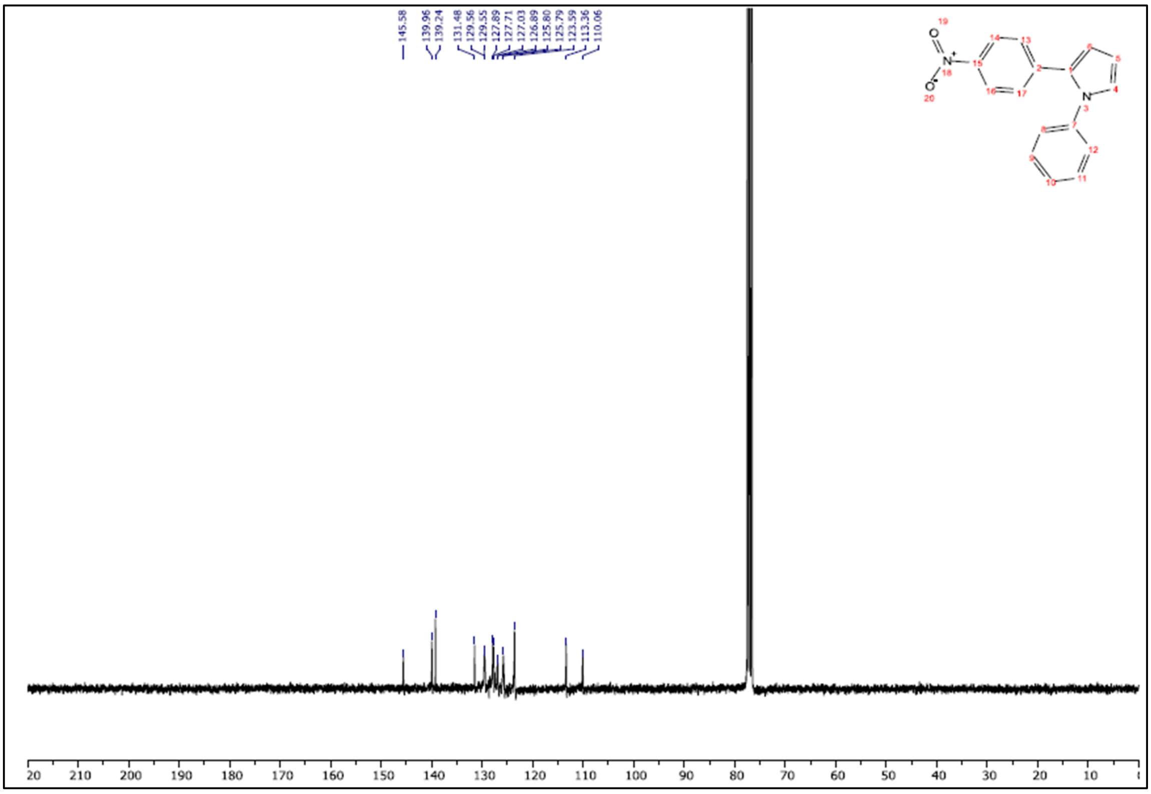

^{1}HNMR of **13c** (300 MHz in $CDCl_3$)

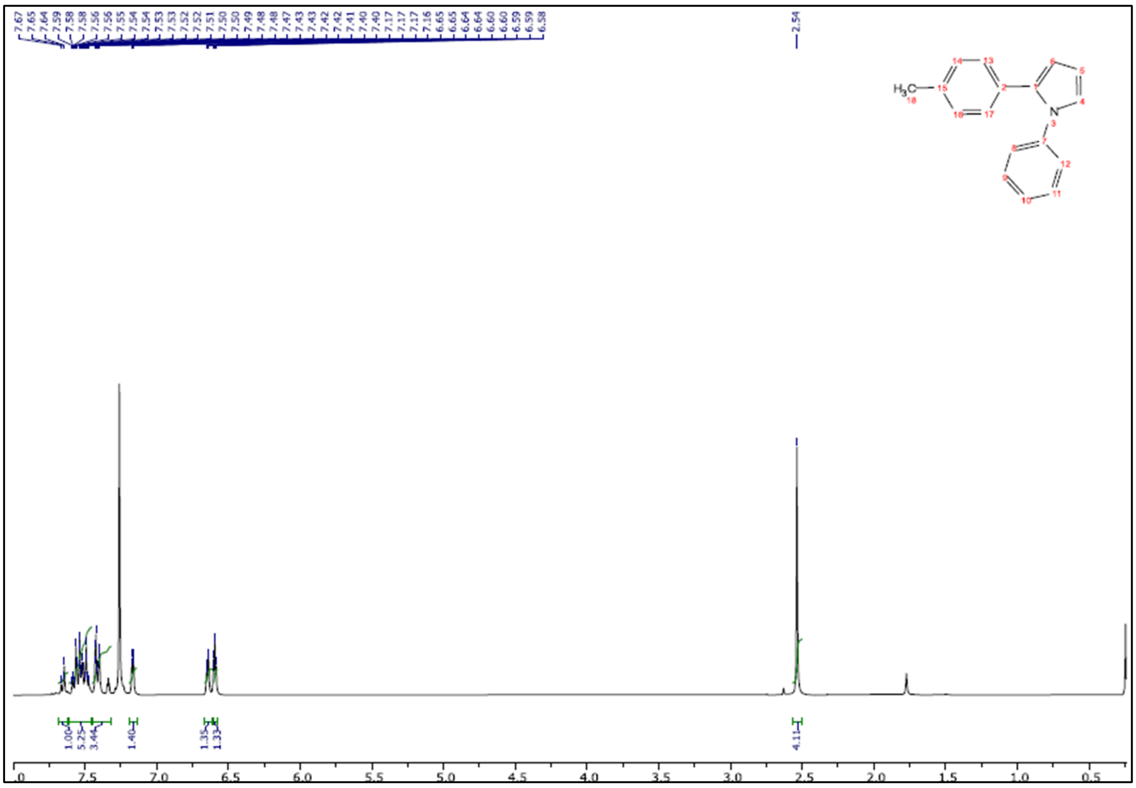

^{13}CNMR of **13c** (75 MHz in $CDCl_3$)

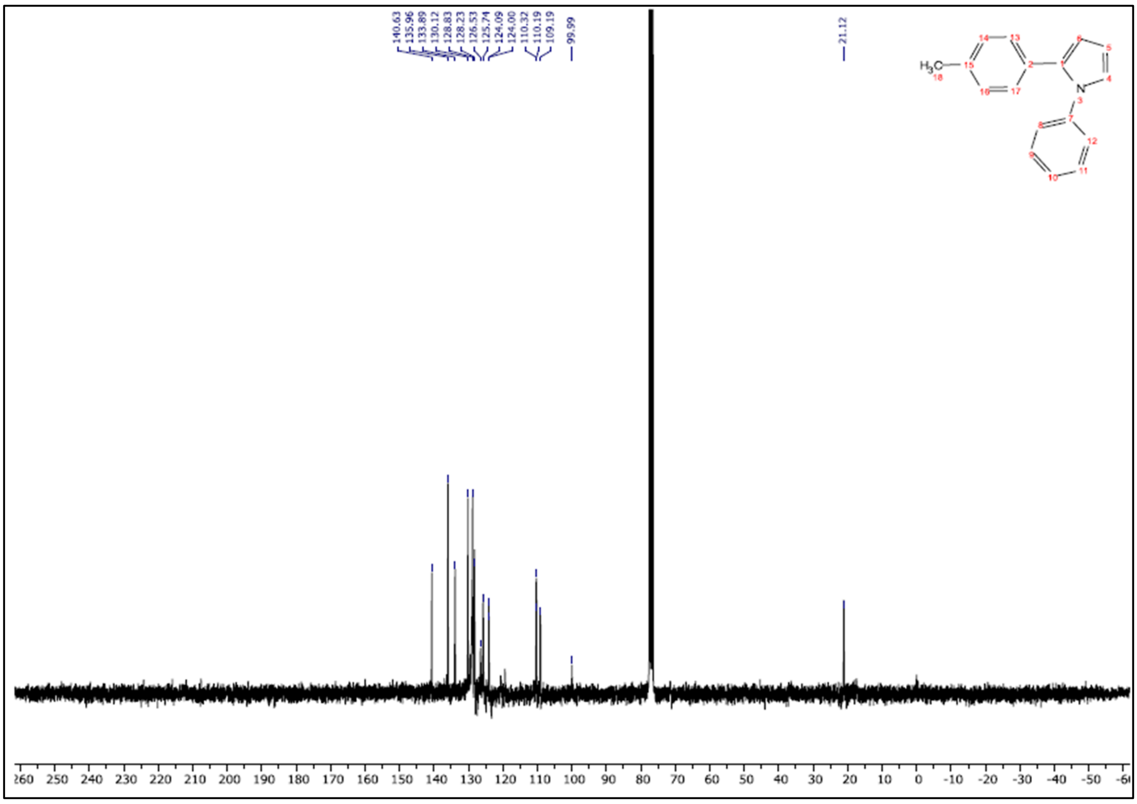

^{1}HNMR of **13d** (300 MHz in $CDCl_3$)

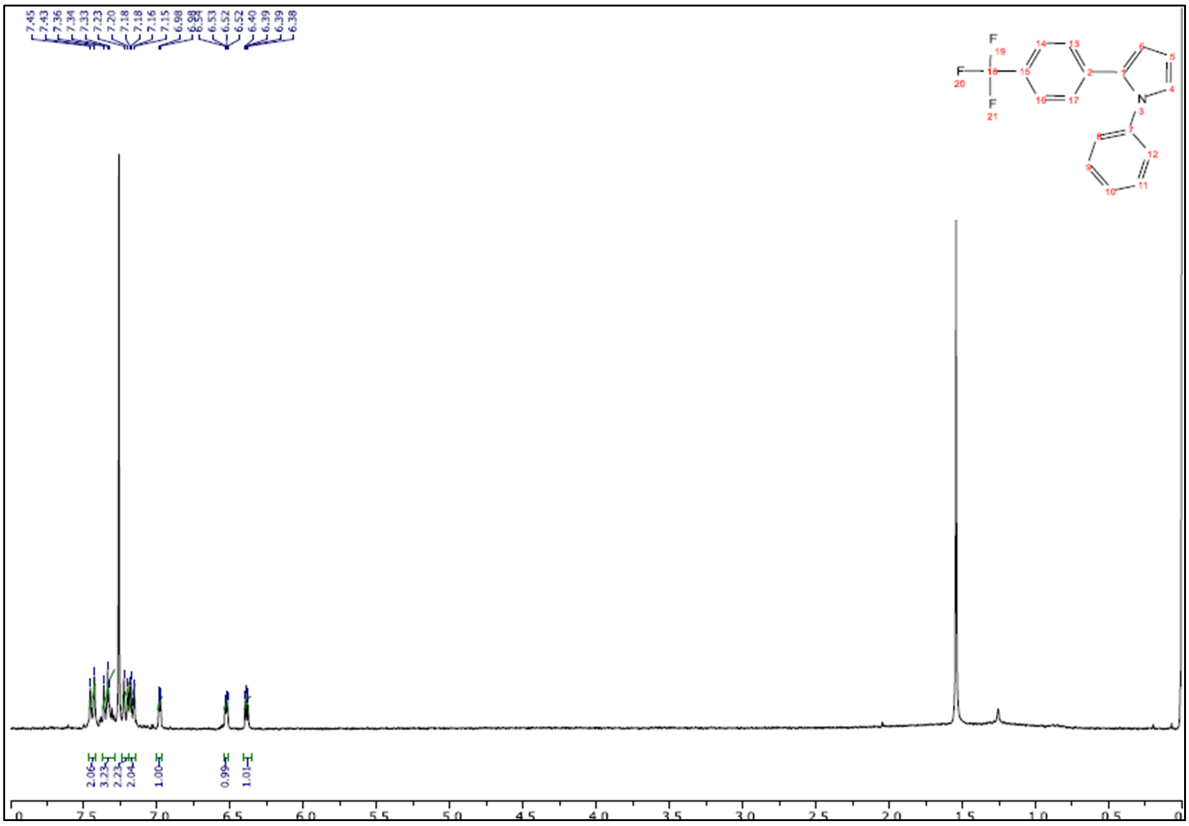

^{13}CNMR of **13d** (75 MHz in $CDCl_3$)

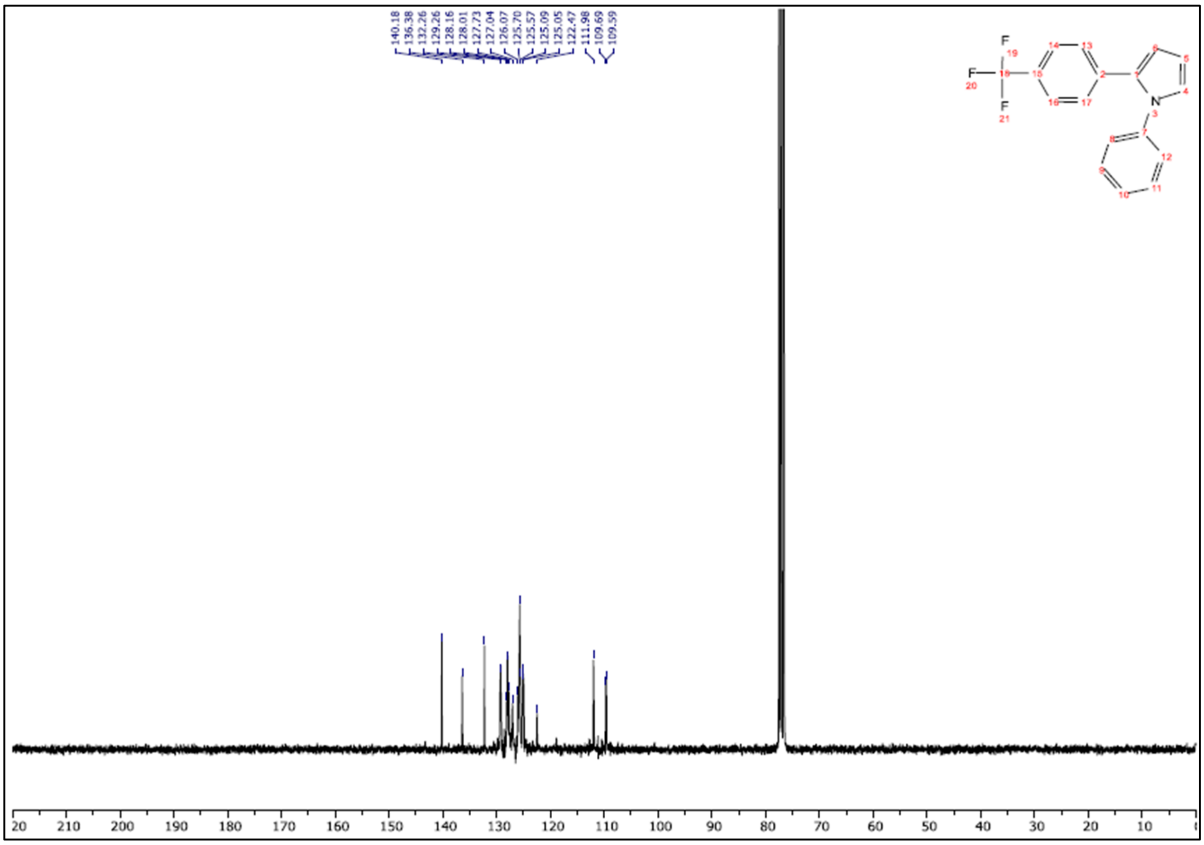

^{1}HNMR of **13e** (300 MHz in $CDCl_3$)

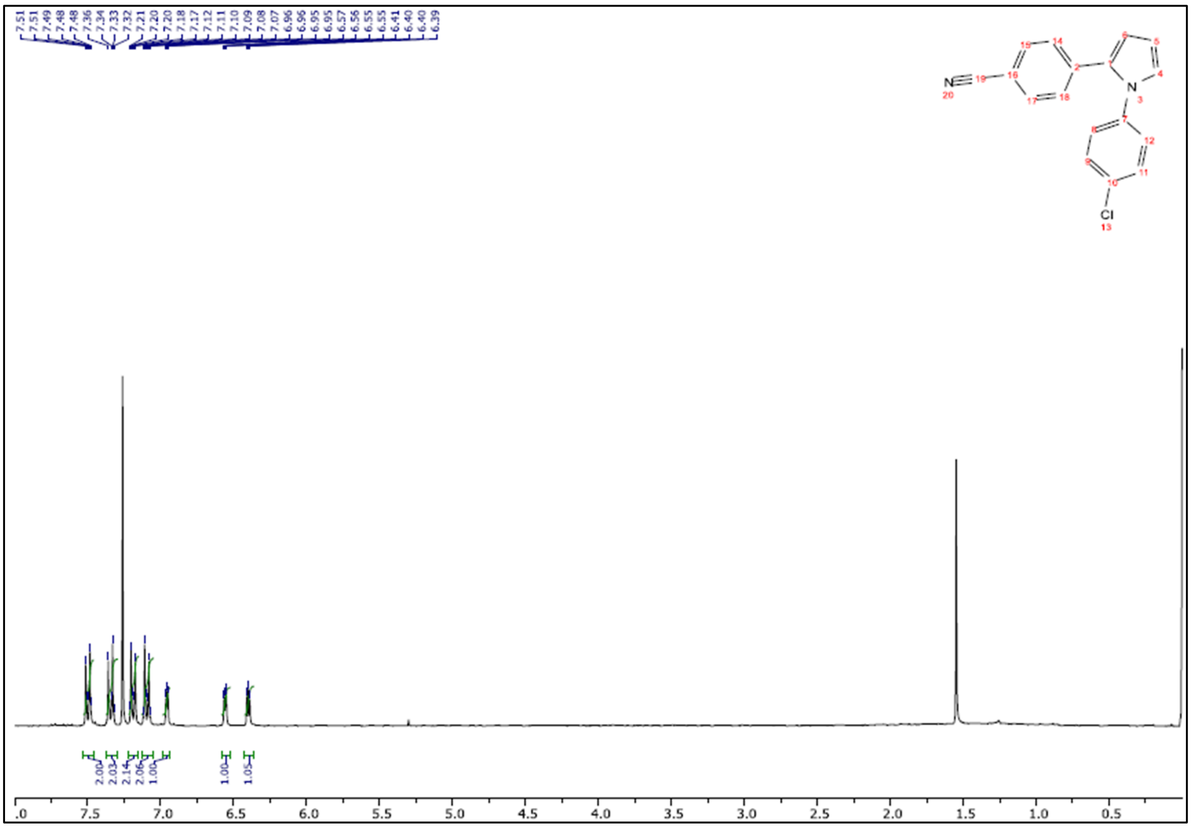

^{13}CNMR of **13e** (75 MHz in $CDCl_3$)

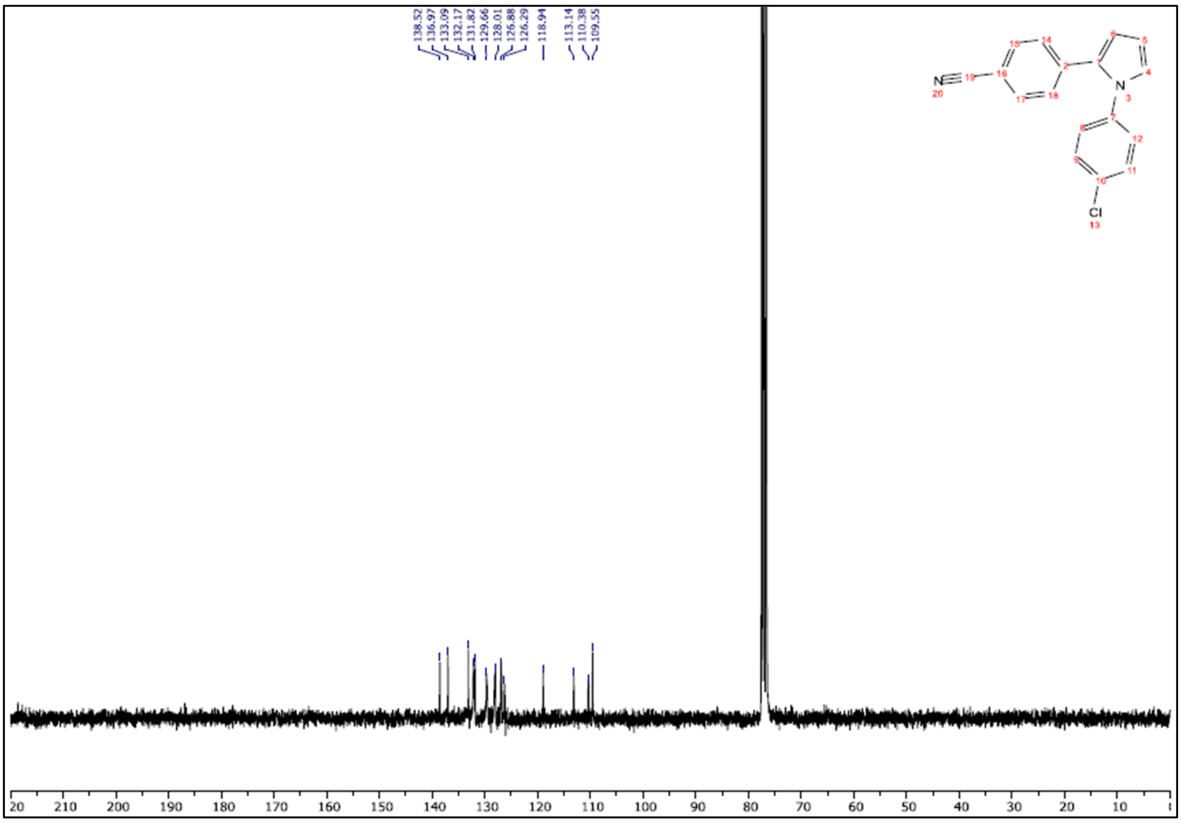

^{1}HNMR of **13f** (300 MHz in $CDCl_3$)

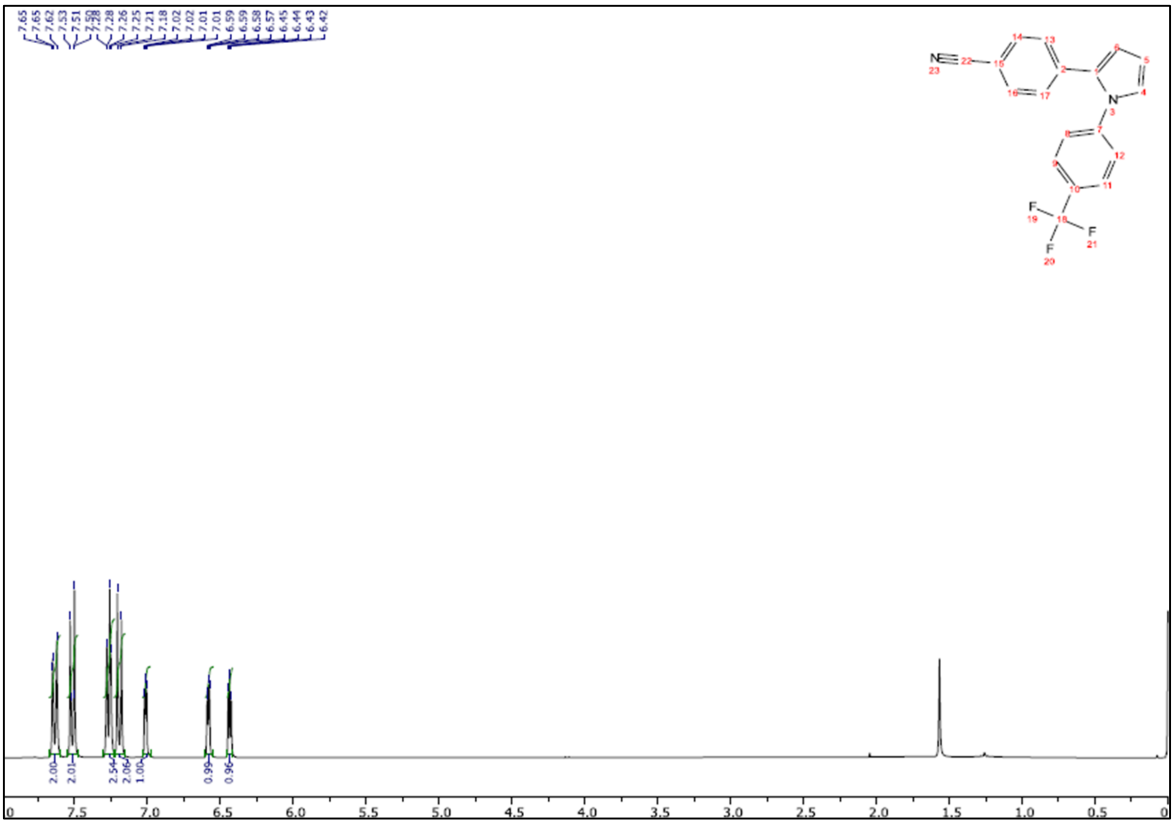

^{13}CNMR of **13f** (75 MHz in $CDCl_3$)

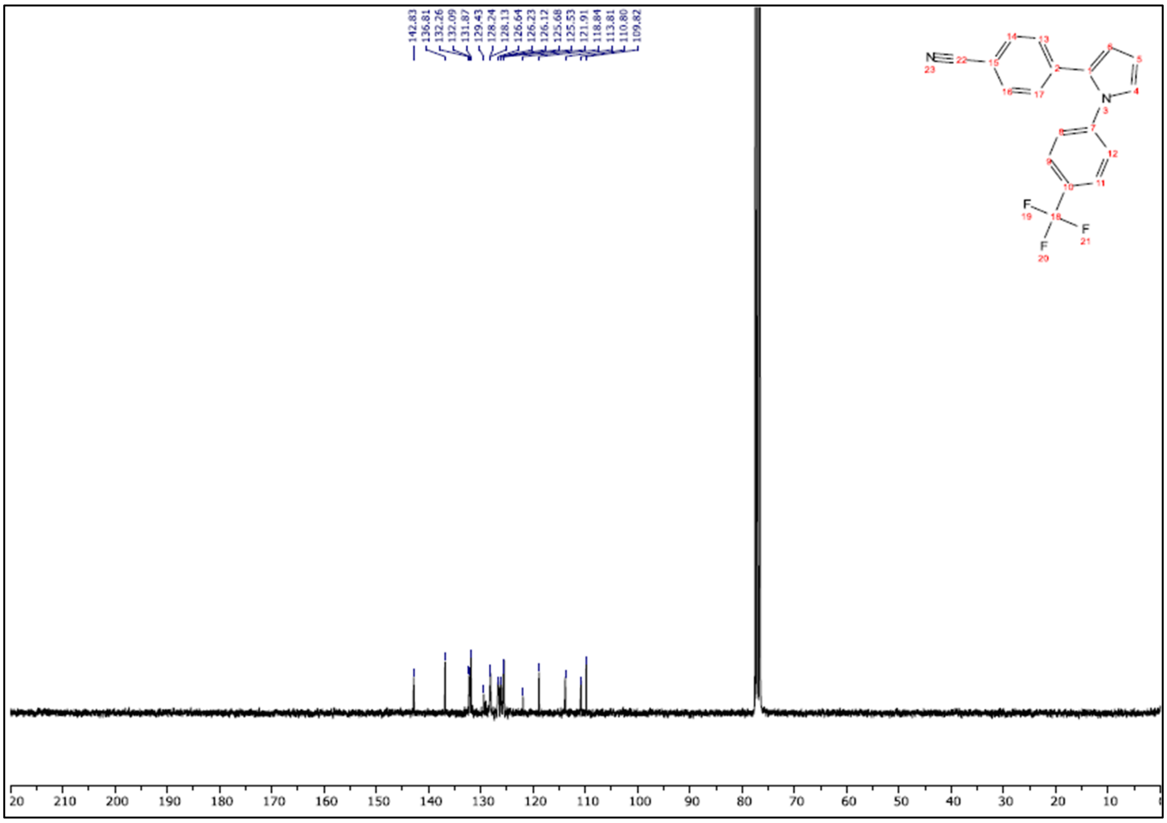

^{1}HNMR of **13g** (300 MHz in $CDCl_3$)

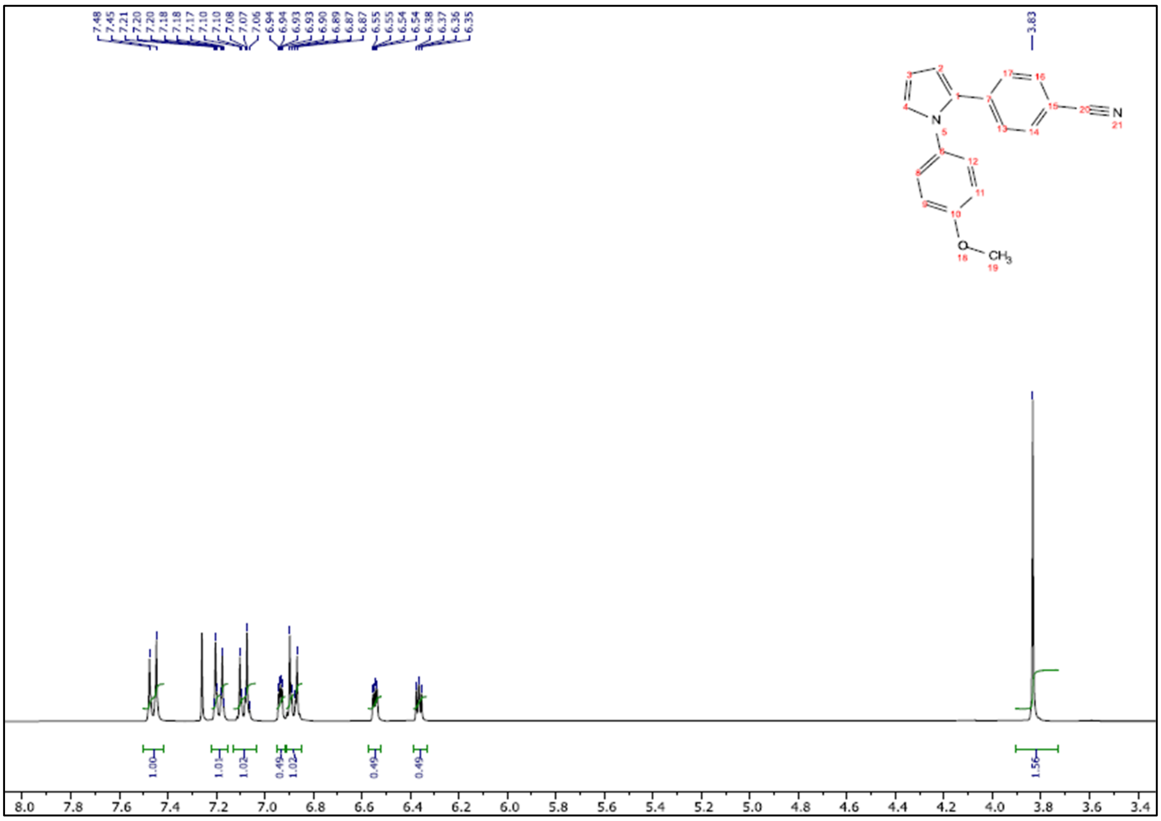

^{13}CNMR of **13g** (75 MHz in $CDCl_3$)

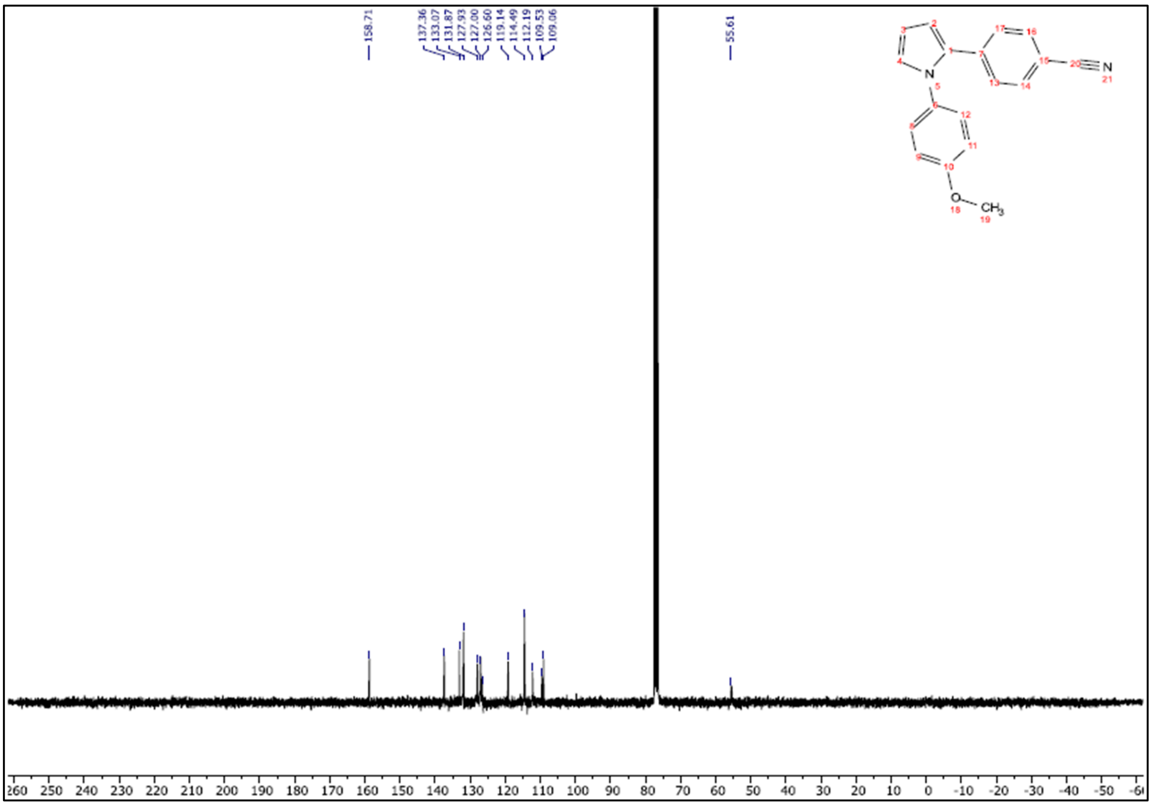

^{1}HNMR of **4a** (500 MHz in $CDCl_3$)

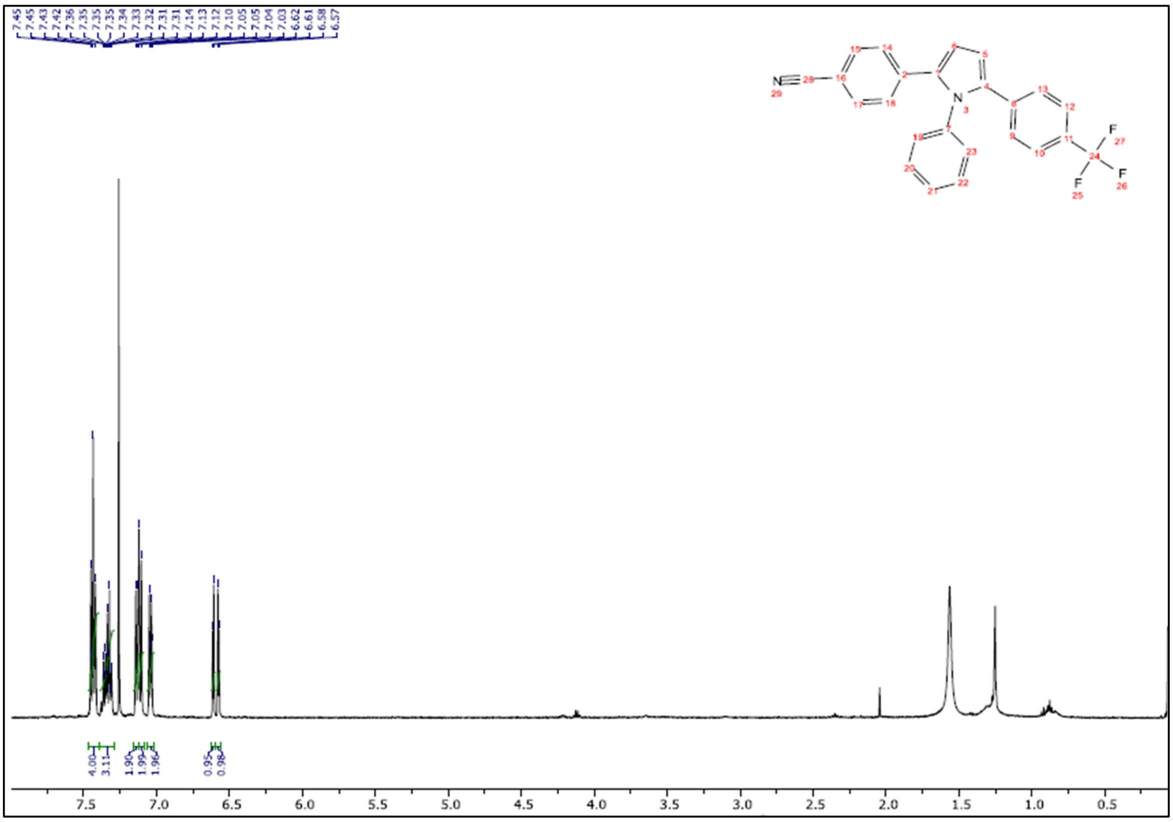

^{13}CNMR of **4a** (126 MHz in $CDCl_3$)

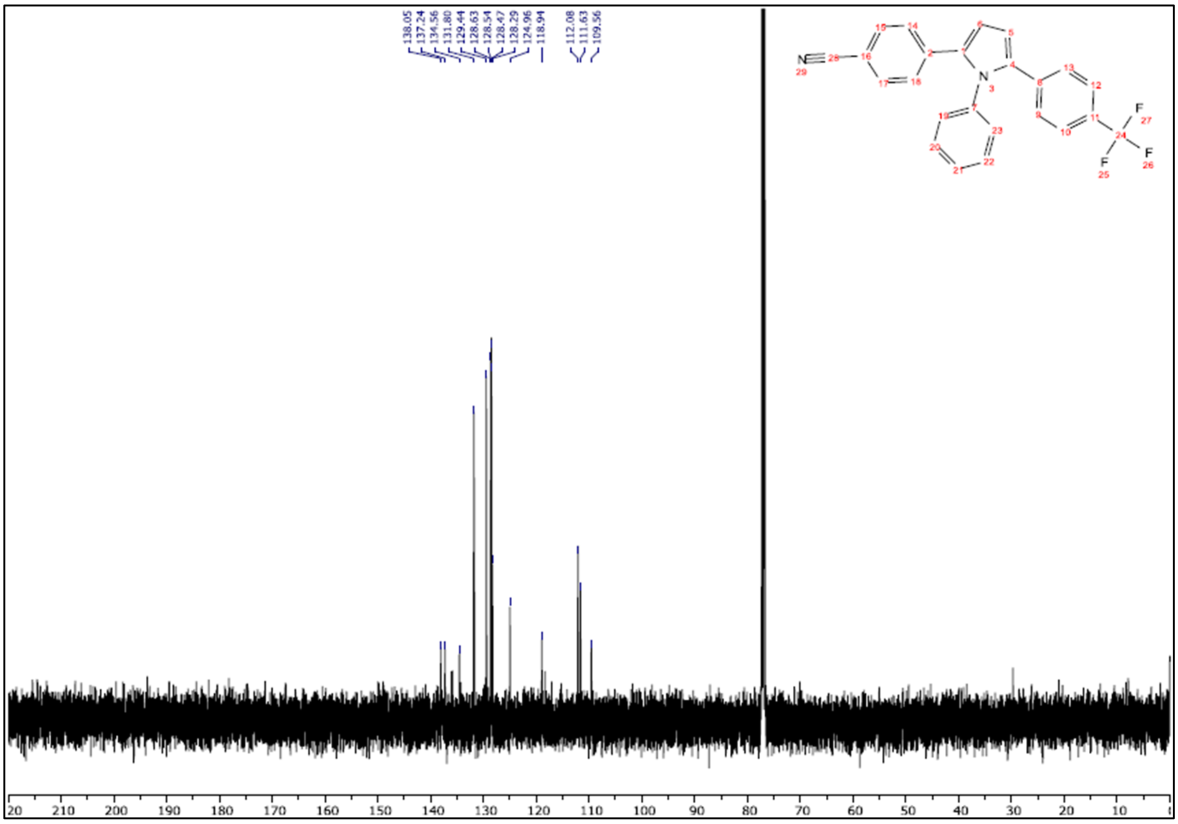

^{1}HNMR of **4b** (500 MHz in $CDCl_3$)

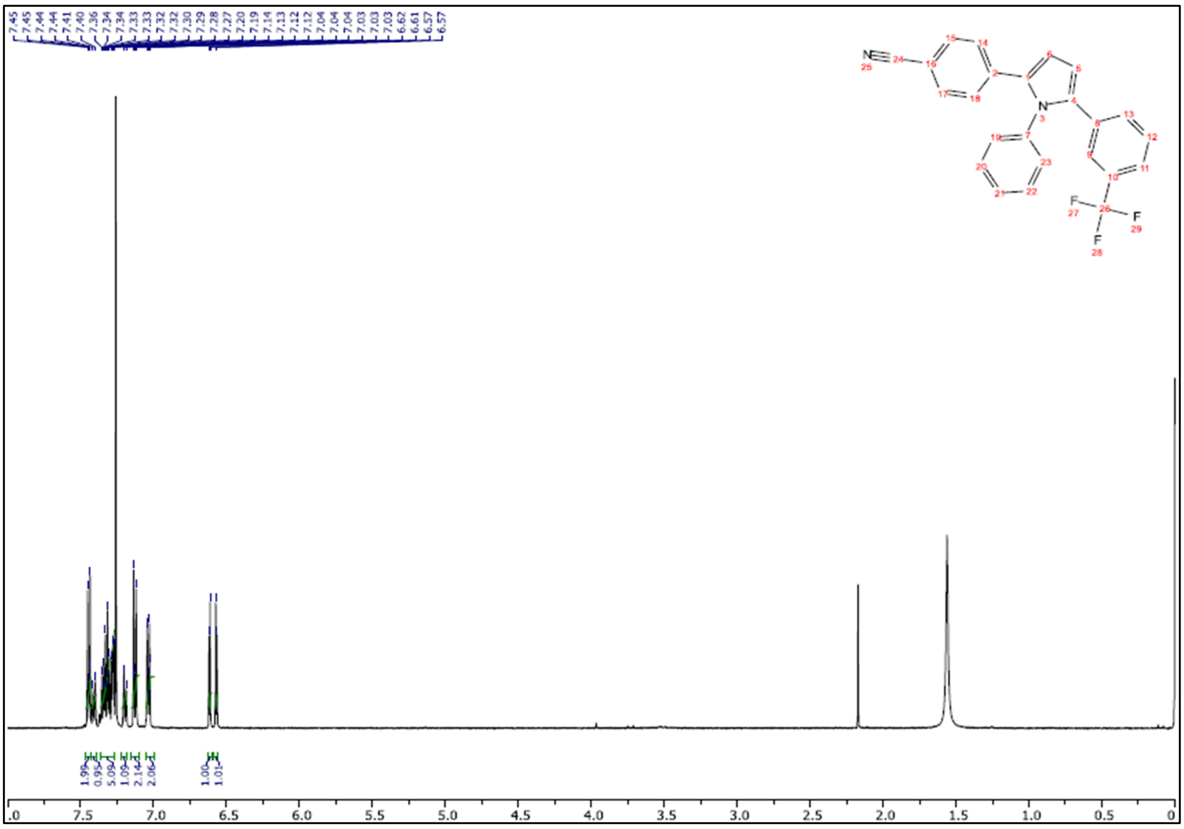

^{13}CNMR of **4b** (126 MHz in $CDCl_3$)

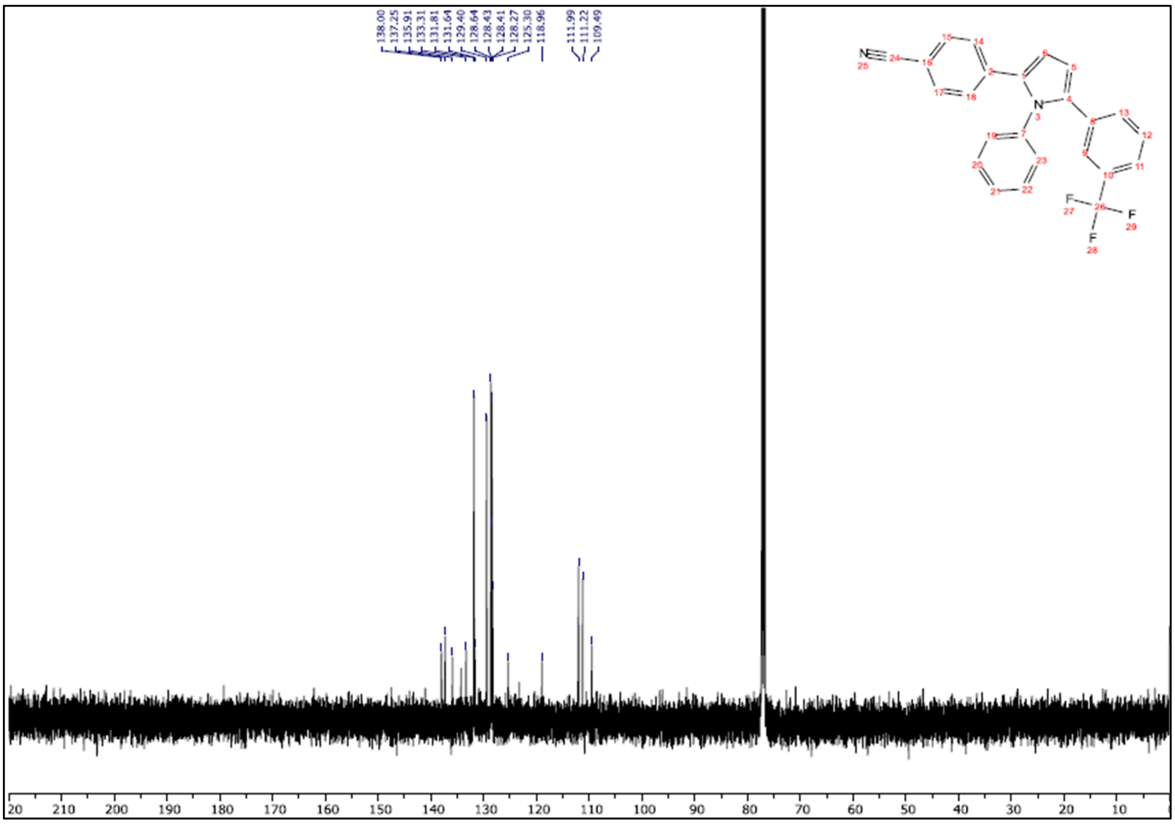

^{1}HNMR of **4c** (500 MHz in $CDCl_3$)

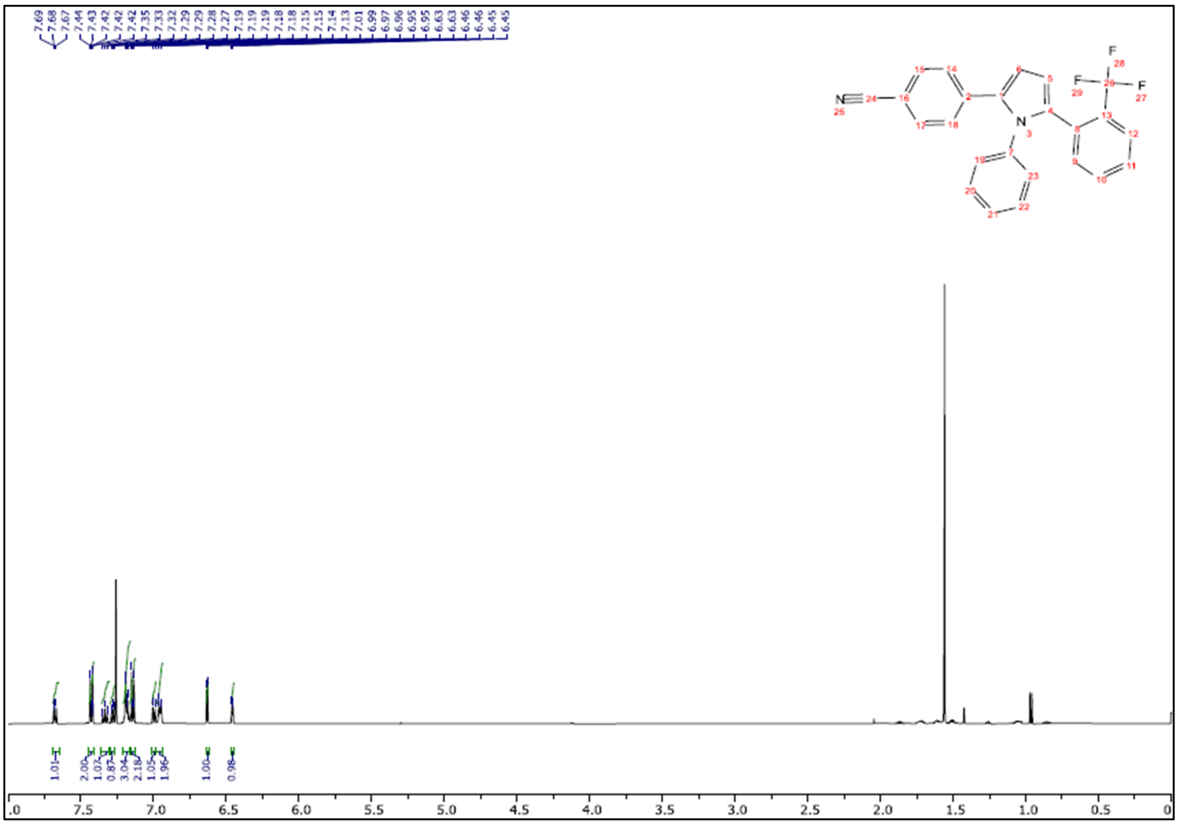

^{13}CNMR of **4c** (126 MHz in $CDCl_3$)

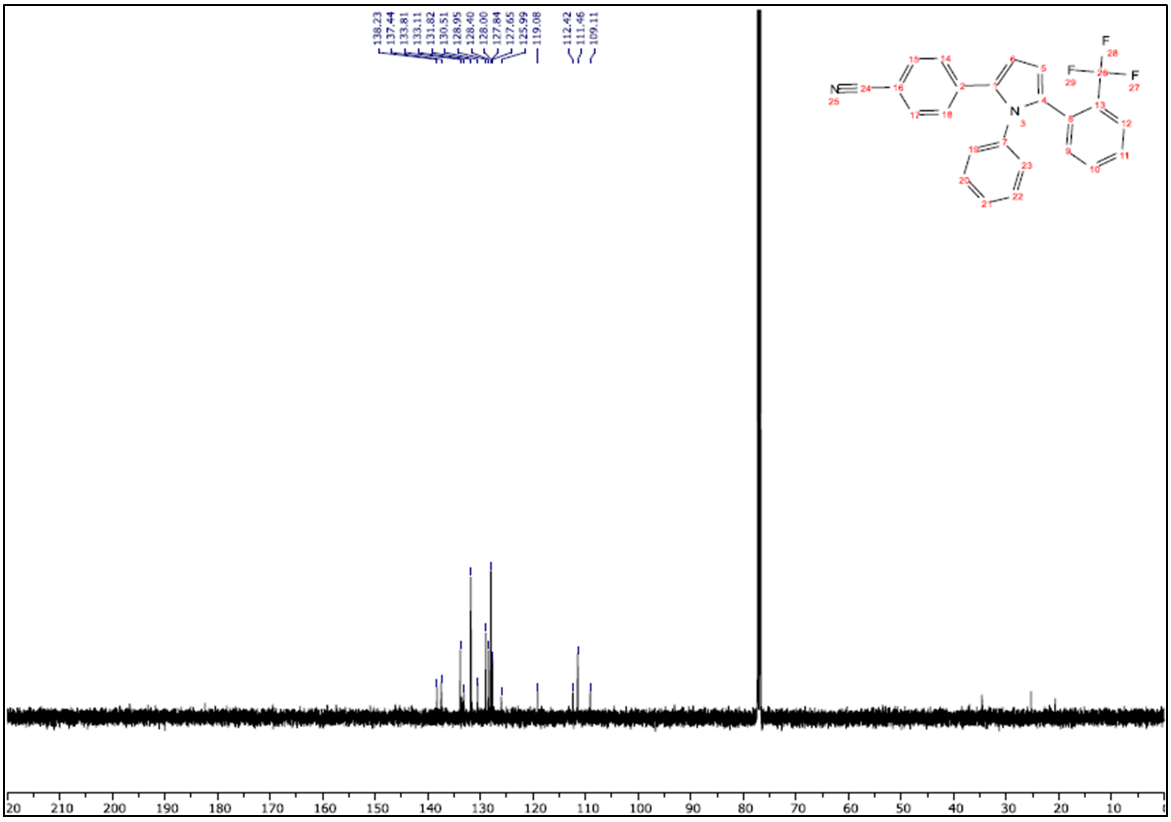

^{1}HNMR of **4d** (500 MHz in $CDCl_3$)

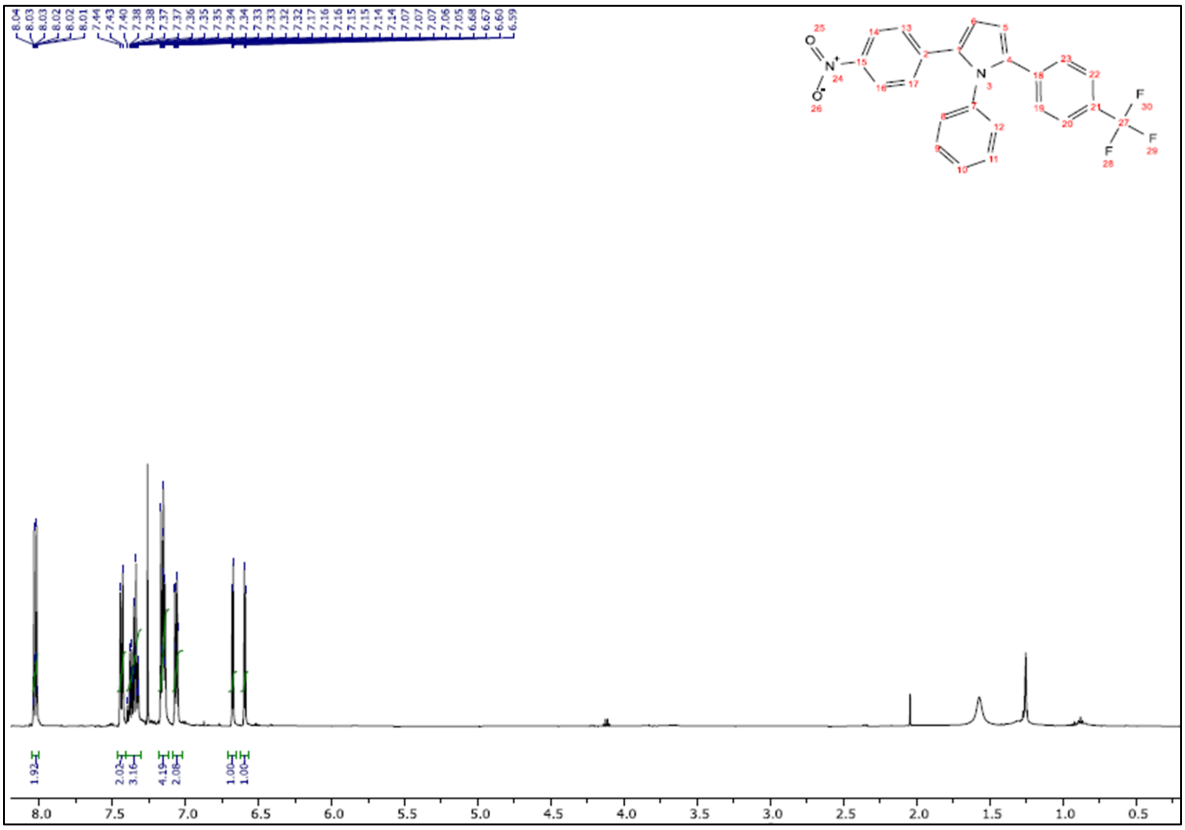

^{13}CNMR of **4d** (126 MHz in $CDCl_3$)

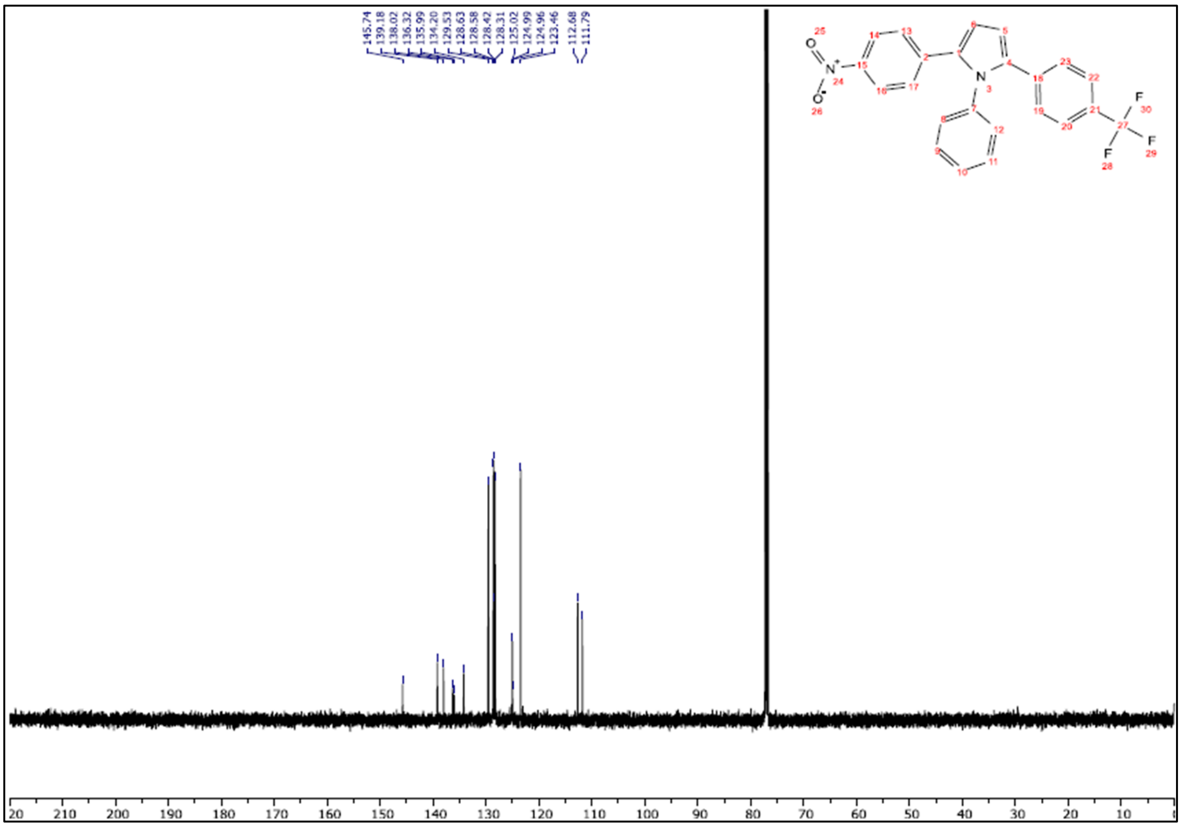

^{1}HNMR of **4e** (500 MHz in $CDCl_3$)

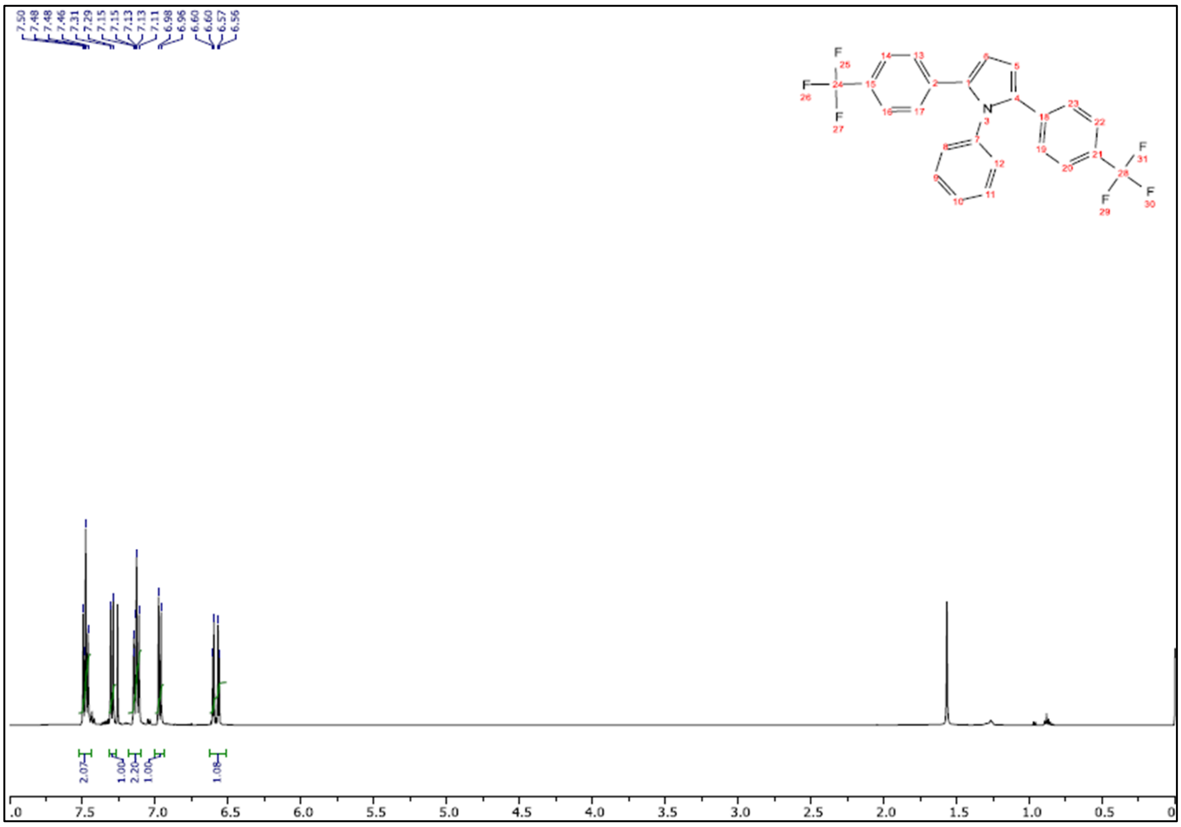

^{13}CNMR of **4e** (126 MHz in $CDCl_3$)

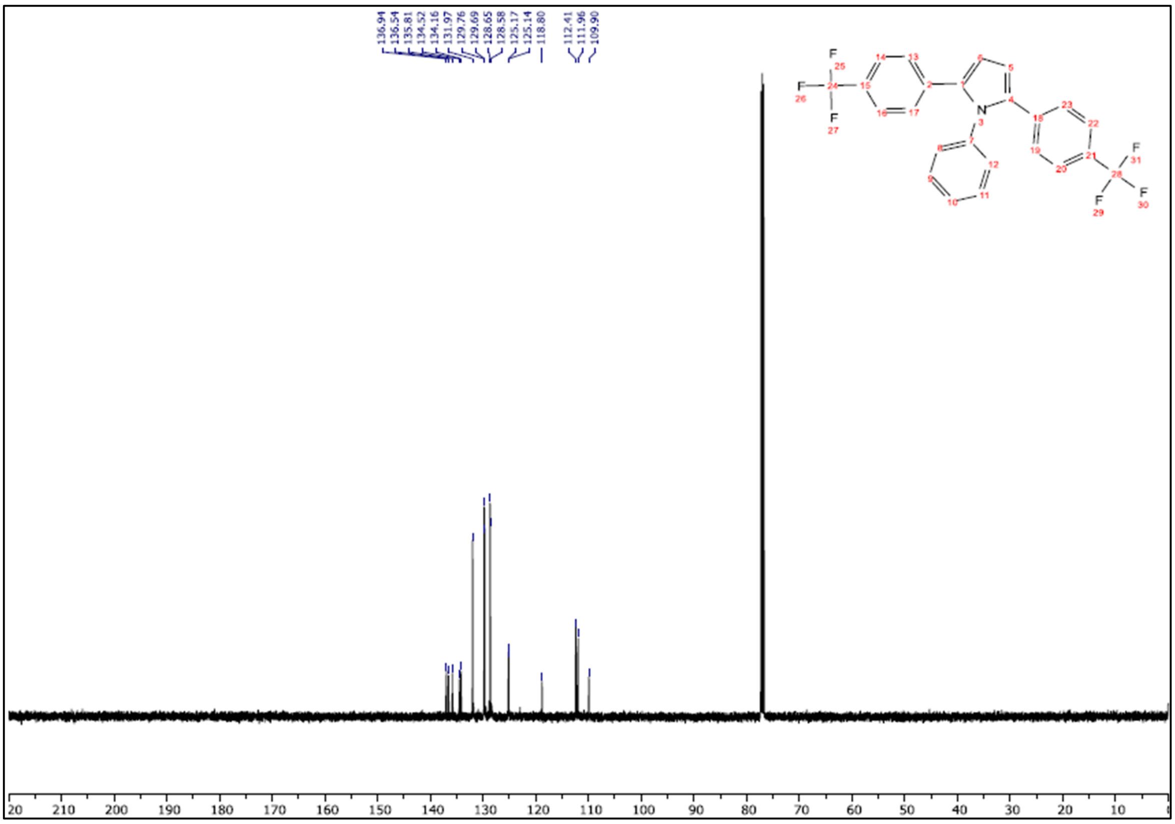

^{1}HNMR of **4f** (500 MHz in $CDCl_3$)

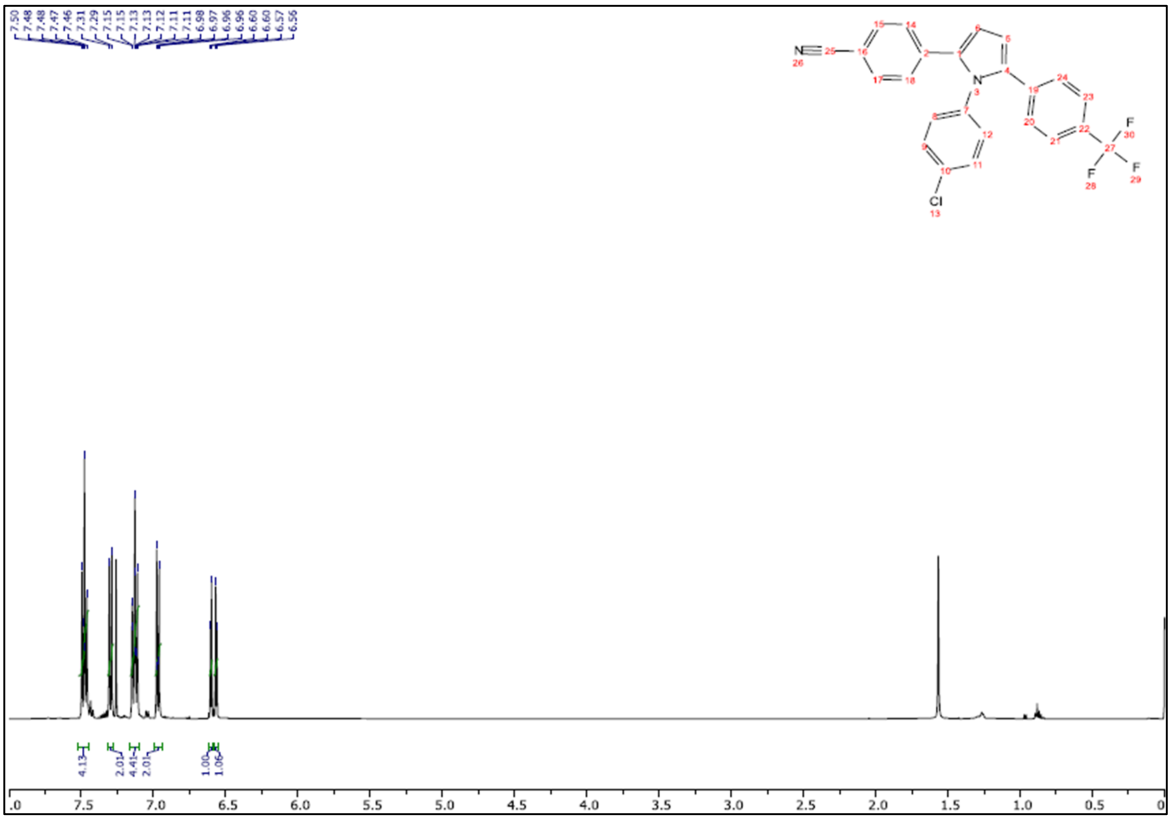

^{13}CNMR of **4f** (126 MHz in $CDCl_3$)

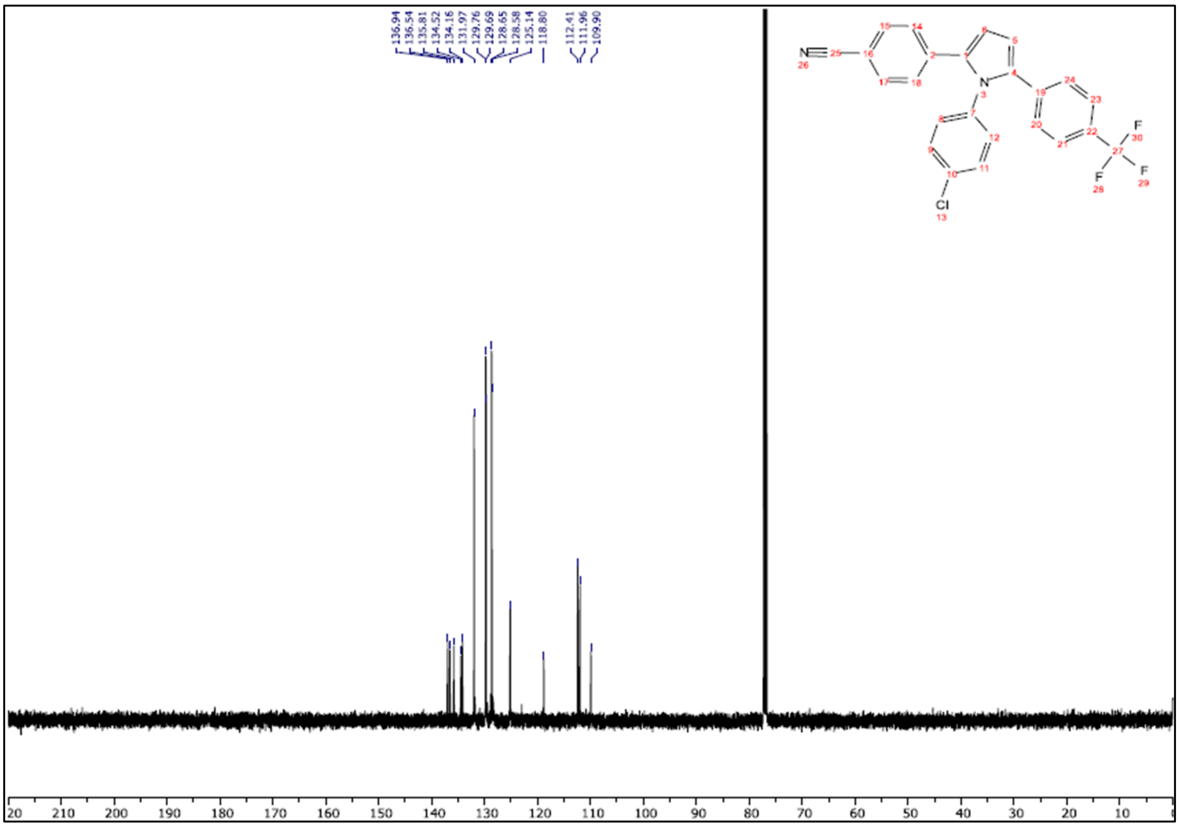

^{1}HNMR of **4g** (500 MHz in $CDCl_3$)

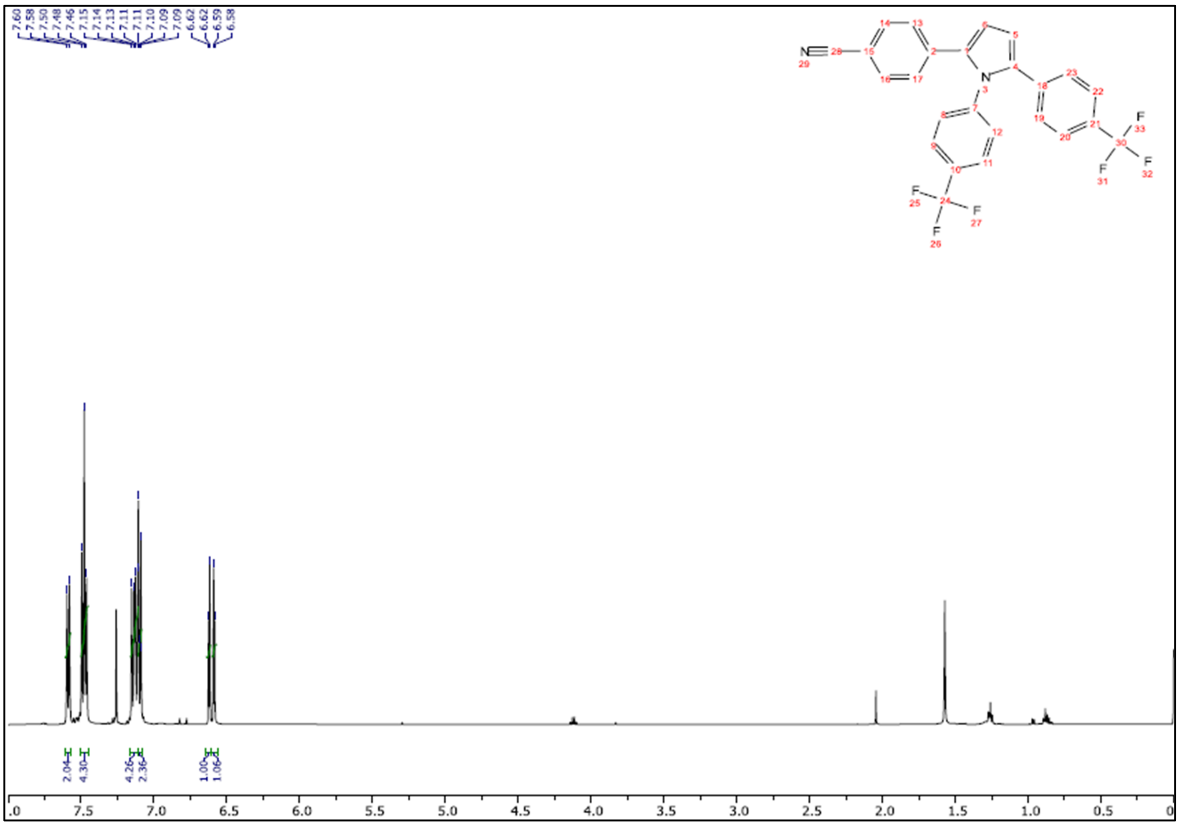

^{13}CNMR of **4g** (126 MHz in $CDCl_3$)

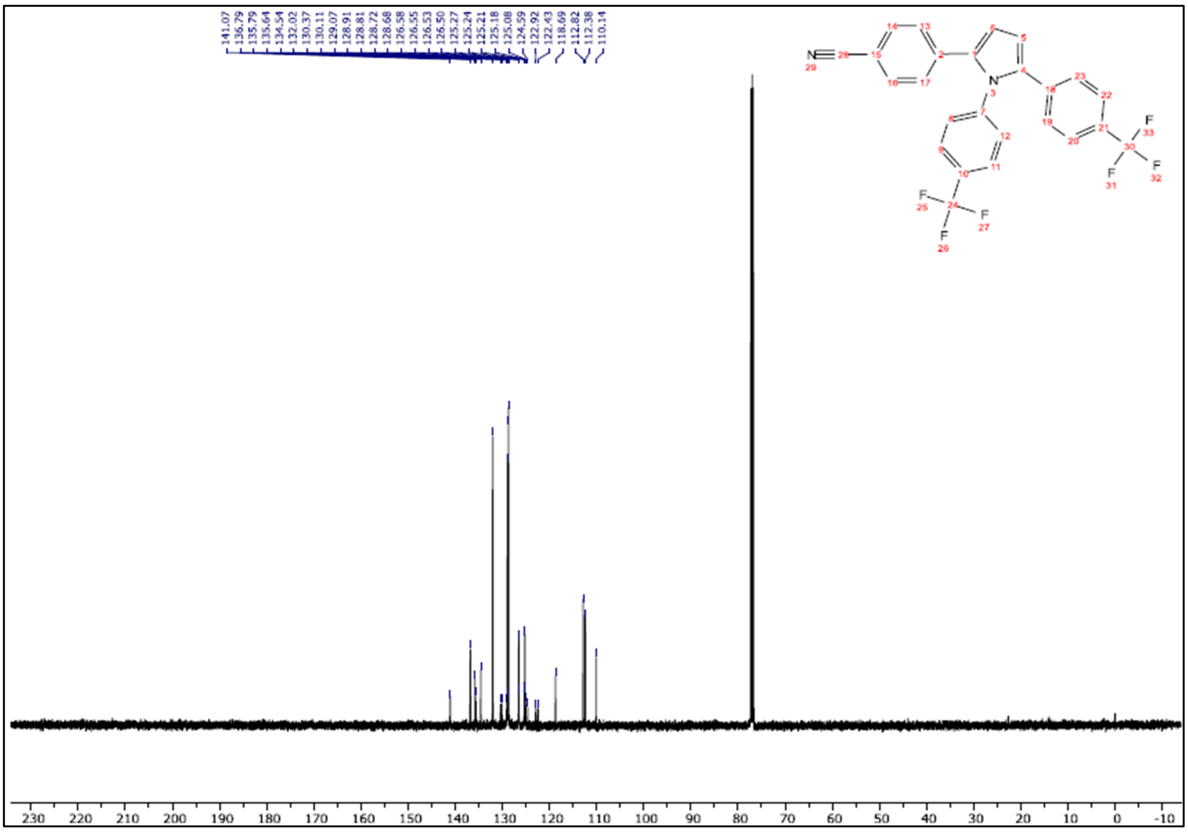

^{1}HNMR of **4h** (500 MHz in $CDCl_3$)

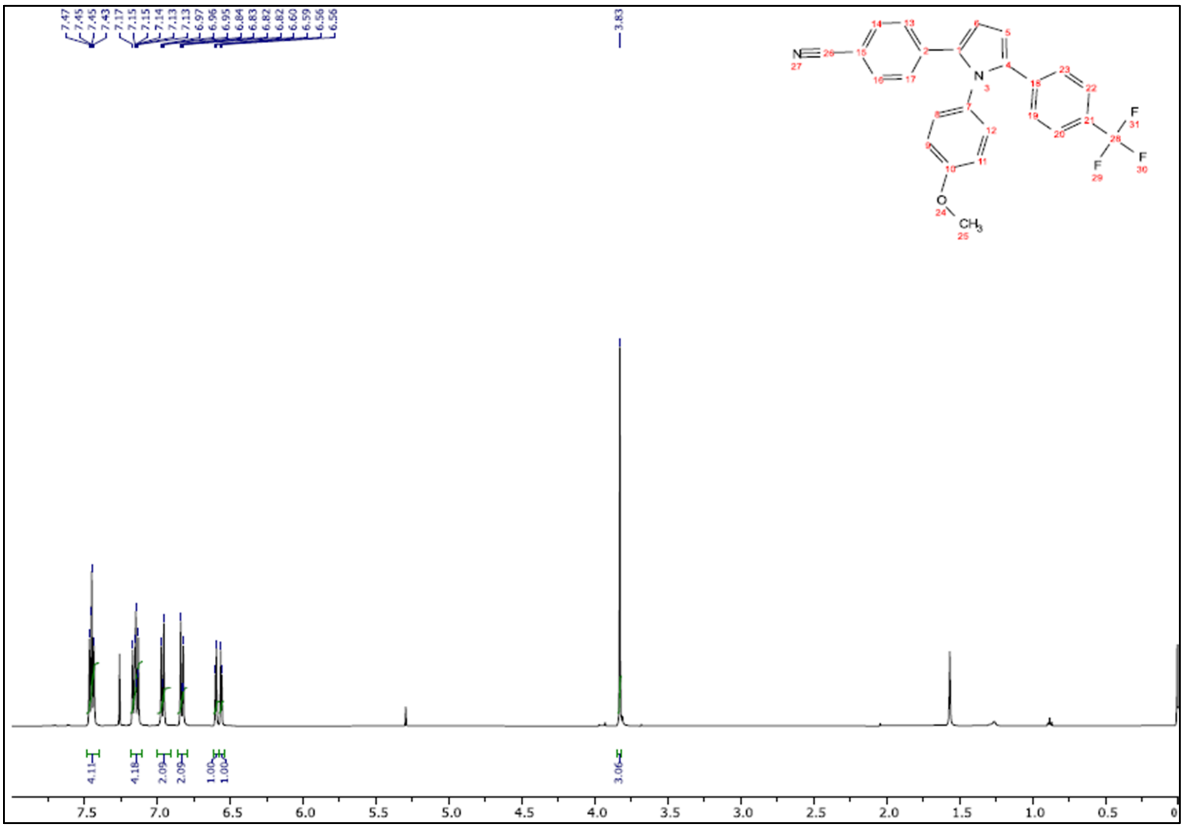

^{13}CNMR of **4h** (126 MHz in $CDCl_3$)

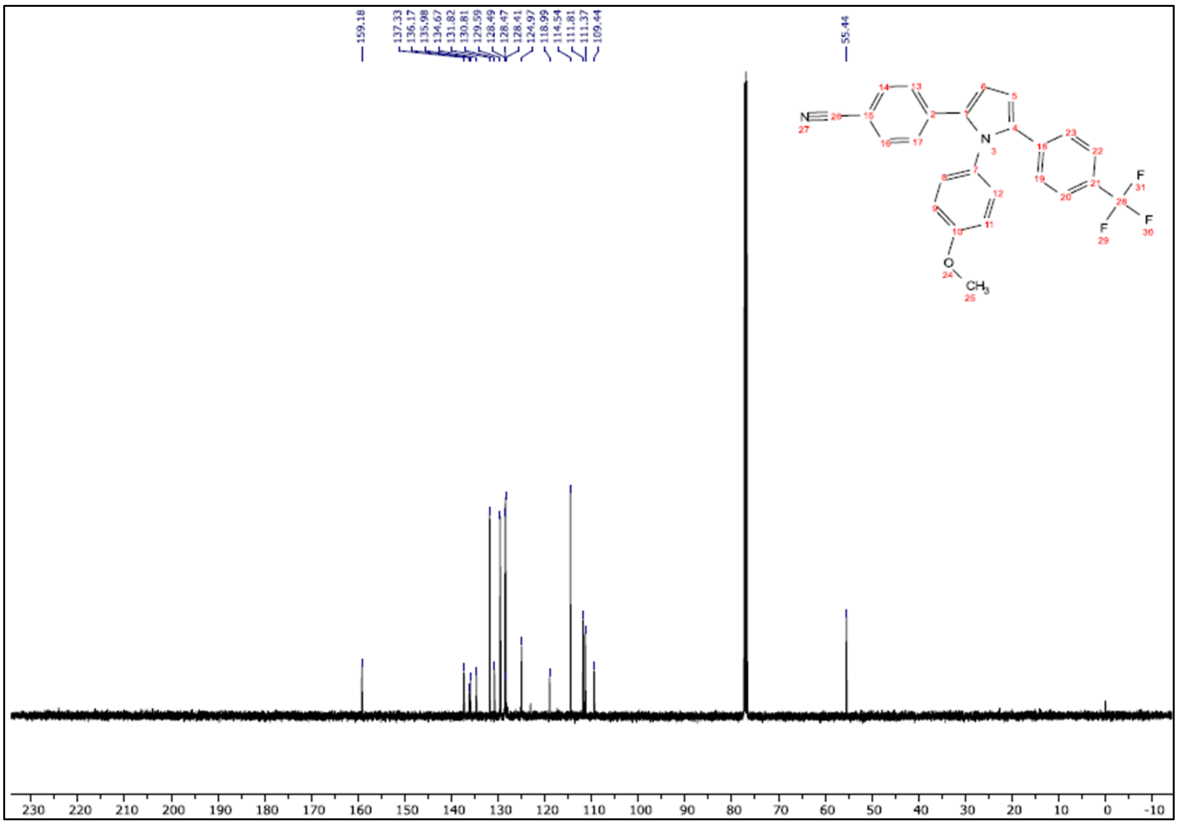

Supplement: Supplementary file 1 [file molecules-31-00986-s001.zip › molecules-4156495-supplementary-File S1.pdf]
